# Estimating and Using Block Information in the Thurstonian IRT Model

**DOI:** 10.1007/s11336-023-09931-8

**Published:** 2023-08-28

**Authors:** Susanne Frick

**Affiliations:** 1 University of Mannheim, Mannheim, Germany; 2 TU Dortmund University, Dortmund, Germany

**Keywords:** multidimensional forced-choice, Thurstonian IRT model, information, standard errors, automated test assembly

## Abstract

Multidimensional forced-choice (MFC) tests are increasing in popularity but their construction is complex. The Thurstonian item response model (Thurstonian IRT model) is most often used to score MFC tests that contain dominance items. Currently, in a frequentist framework, information about the latent traits in the Thurstonian IRT model is computed for binary outcomes of pairwise comparisons, but this approach neglects stochastic dependencies. In this manuscript, it is shown how to estimate Fisher information on the block level. A simulation study showed that the observed and expected standard errors based on the block information were similarly accurate. When local dependencies for block sizes $>\,2$ were neglected, the standard errors were underestimated, except with the maximum a posteriori estimator. It is shown how the multidimensional block information can be summarized for test construction. A simulation study and an empirical application showed small differences between the block information summaries depending on the outcome considered. Thus, block information can aid the construction of reliable MFC tests.

**Supplementary Information:**

The online version contains supplementary material available at 10.1007/s11336-023-09931-8.

Test constructors aim to develop tests that provide reliable and valid measurement of their constructs of interest. Most personality tests employ rating scales (e.g., strongly disagree, disagree, etc.) for this purpose, but responses to rating scales are potentially biased, for example, by response styles (Henninger & Meiser 2020; Krosnick 1999; Wetzel et al. 2016). As an alternative, the multidimensional forced-choice (MFC) format has been increasing in popularity. In the MFC format, several items measuring different attributes are presented simultaneously in blocks. The respondent’s task is then to rank the items (see Fig. 1 for an example) or select the ones that they prefer the most and/or the least. This research is concerned with the former, which is called full ranking.^1^

**Fig. 1:**
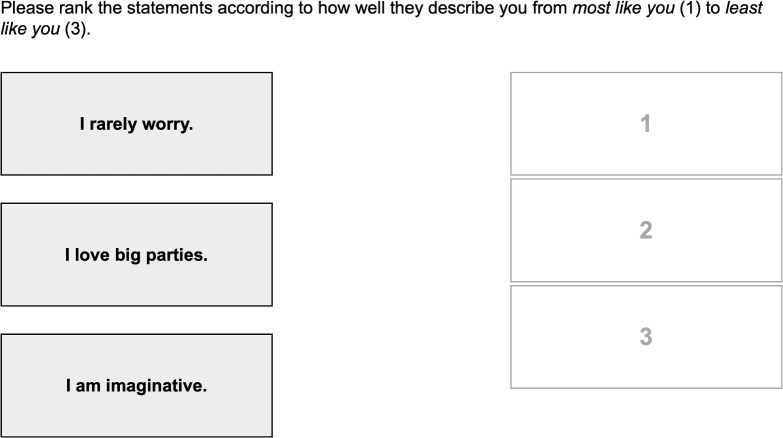
Example of the multidimensional forced-choice format from the Big Five Triplet (Wetzel & Frick 2020). The first item assesses neuroticism (reverse-coded), the second extraversion, and the third openness.

In comparison with rating scales, the MFC format has the advantage to avoid or reduce several response biases. For example, overall faking is reduced (Cao & Drasgow 2019; Wetzel et al. 2021), and uniform response biases, such as halo effects, are avoided (Brown et al. 2017); for an overview, see Brown & Maydeu-Olivares (2018a).

As interest in the MFC format increases, it is important for test constructors to know how to construct such tests. However, test construction is more complicated than with rating scales since the combination of items into blocks can affect the item properties. This is because in the MFC format, the test taker does not evaluate the items in a block independently but instead must weigh them against each other when deciding how to respond. In other words, the responses given are relative instead of absolute, such as in a rating scale or true–false response format. In line with this, research has found that the measured constructs change slightly when the same items are presented in an MFC format versus a rating scale format (Guenole et al. 2018; Wetzel & Frick 2020). Further, item desirability is evaluated differently in the context of MFC blocks than it is for single-stimulus items (Feldman & Corah 1960; Hofstee 1970). More specifically, item parameters from item response theory (IRT) models were found to differ depending on which items were combined into blocks (Lin & Brown 2017). Thus, item properties are dependent on the specific combination of items that form a block. Re-assembling items to form new blocks bears the danger that the item properties change and the test does not work as expected.

Therefore, MFC blocks should be treated as fixed units during test construction and not be re-assembled. The test construction process then becomes a process of selecting blocks instead of items. To quantify how each block contributes to measurement precision, information on the block level (henceforth termed *block information*) comes as a natural metric, because it summarizes all the item parameters within a block.

For MFC tests with ideal-point items—that is, where the preference for an item is highest at a certain trait level and decreases as the distance from it increases—block information can be calculated on the basis of the generalized graded unfolding model for rank responses (Joo et al. 2018). It has been shown that this approach can be used to construct computerized adaptive tests (Joo et al. 2020). However, most tests employ dominance items, where the preference for an item increases or decreases monotonically as trait levels increase. For MFC tests with dominance items, block information can be derived analytically when a logit link is used as in the multi-unidimensional pairwise preference 2PL model (Kreitchmann et al. 2023; Morillo et al. 2016). This has been used in computerized adaptive testing (Kreitchmann et al. 2023).

The Thurstonian IRT model (Brown & Maydeu-Olivares 2011) has become the most popular and widely applicable IRT model for MFC data. The Thurstonian IRT model can incorporate different block sizes and different response instructions, such as ranking all items in a block or picking one of them. In the Thurstonian IRT model, for block size two (pairs of items), information can be analytically derived (e.g., Brown & Maydeu-Olivares 2018b; Bürkner 2022). For block sizes larger than two, block information must be numerically approximated (Yousfi 2018) because the integrals involved are not analytically tractable (Genz & Bretz 2002, 2009). Since there is no formula for Thurstonian IRT block information, it can also not be linearly approximated as is often done with multidimensional information (van der Linden 2005). More precisely, both obtaining the response probability (Eq. 3) and obtaining its Hessian (Eq. 7) involve numerical approximation. Thus, block information is essentially an estimate. Therefore, in order to evaluate whether block information can be used for test construction, it is crucial to examine the accuracy of its estimation.

The aims of this paper are to evaluate how well the numerical approximation of block information works (a) on the test level and (b) on the block level in simulation studies and to showcase how to use block information for test construction. On the block level, there is no clear reference point for what constitutes accurate information. Therefore, the first simulation examines the accuracy of standard errors, that is, the inverse of test information (i.e., the sum of the block information). The second simulation simulates the test construction process based on block information. In addition, two aspects relevant for the Thurstonian IRT model motivate the research questions and the design of the simulation studies, namely computational time and multidimensionality.

In the first part, I investigate the accuracy of information on the test level by investigating the accuracy of standard errors. Computing Thurstonian IRT block information is computationally intensive, because it involves two steps of numerical approximation and is not yet easily implemented in standard software. Instead, the latent traits are usually estimated via a pseudo-likelihood that neglects local dependencies (Brown & Maydeu-Olivares 2011). This is both faster and easy to implement. Yousfi (2018, 2020) showed that this procedure does not affect point estimates for Thurstonian IRT traits but their standard errors. However, Yousfi’s (2018; 2020) examination was only theoretical. I extend Yousfi’s theoretical examination by varied trait levels, realistic item parameters, and different estimators. Therefore, the first research question investigated is:

RQ1: Does neglecting local dependencies in the computation of standard errors affect their precision?

Typically, in IRT, standard errors are obtained via taking the expectation over all possible response patterns. However, in practice, standard errors are often obtained based on the observed response pattern only. The latter saves computational time and effort which might be especially worthwhile for the Thurstonian IRT model where information estimation is computationally intensive. Therefore, the second research question is:

RQ2: How accurate are observed versus expected standard errors?

In the second part, it is investigated whether block information is sufficiently accurate to be used in test construction. For this purpose, I simulate the test construction process based on block information and provide an empirical application. Since block information is multidimensional, it can be summarized into a scalar in different ways. I compare different information summaries and algorithms that can be used to assemble MFC tests from fixed block compositions. Therefore, the research question for the second part is:

RQ3: How well do different information summaries perform for selecting blocks in test construction?

In the following, before presenting the simulation studies, I first introduce the Thurstonian IRT model more formally and present formulas (as far as they are available) for calculating block information when considering and neglecting local dependencies.

An R package implementing the block information estimation, the information summaries and the automated test assembly algorithms is available at GitHub: https://github.com/susanne-frick/MFCblockInfo. The R-code for running and analyzing the simulations and the simulation results are available from the same GitHub repository.

## The Thurstonian IRT Model

In the Thurstonian IRT model, for each item, there is a latent response tendency called utility. The utility *t* for person *j* on item *i* is a linear function of a latent trait $\theta _j$:

1 $$\begin{aligned} t_{ji} = \mu _i + \lambda _i\theta _j + \varepsilon _{ji} \end{aligned}$$

where $\mu _i$ denotes the item intercept, $\lambda _i$ the item loading, and $\varepsilon _{ji}$ the error term. In the following, vectors and matrices are indicated by boldface notation. The latent traits are assumed to be multivariate normally distributed: $\varvec{\Theta } \sim N(\mathbf {M_\theta }, \mathbf {\Sigma })$, and the vectors of item errors are independently normally distributed: $\varvec{\varepsilon }_i \sim N(0,\psi _i^2)$.

According to Thurstone’s law of comparative judgment (Thurstone 1927, 1931), participants order the items within each block according to the magnitude of their utilities.

### Genuine Likelihood

To express this mathematically, first, let $t^{\star }_{ji}$ denote the systematic utilities $t^{\star }_{ji} = \mu _i + \lambda _i\theta _j$, that is, without the error term. Second, within each block indexed by *k*, for block size *B*, vectors of utilities ${\varvec{t}}^{\star }_{jk} = (t^{\star }_{j1} \dots t^{\star }_{jB})'$ and error variances $\varvec{\psi }_k^2 = (\psi _1^2 \dots \psi _B^2)'$ are sorted in descending order, according to the selected rank order. The possible rank orders (i.e., the $R=B!$ permutations of the *B* items) are indexed by *r*. Hence, the ordered utilities are denoted as ${\varvec{t}}^{\star }_{jkr}$. Third, differences between consecutive utilities $\textit{\textbf{A t}}^{\star }_{jkr}$ are obtained by employing a comparison matrix ${\textbf{A}}$ of size $(B-1) \times B$. For example, if block size $B=3$:

2 $$\begin{aligned} {\varvec{A}}_{B=3} = \begin{pmatrix} 1 &{} -1 &{} 0 \\ 0 &{} 1 &{} -1 \end{pmatrix}. \end{aligned}$$

With the utilities sorted in a descending order, each difference between two consecutive utilities is positive. Therefore, the probability of selecting rank order *r* is the area under the multivariate normal density of utilities where this applies (Yousfi 2020):

3 $$\begin{aligned} P(X_{jk}=r | \varvec{\theta }_{j}) = \int _{{\textbf{0}}}^{\mathbf {\infty }}{N}_{\varvec{A t}^{\star }_{jkr}, \varvec{A {{\,\textrm{diag}\,}}(\psi _{kr}^2) A'}}\left( \varvec{\zeta }\right) d\varvec{\zeta } \end{aligned}$$

Hence, there are $B-1$ nested integrals, one for each consecutive comparison. There is no analytical solution to this multiple integral but it can be numerically approximated (Genz & Bretz 2002; Genz 2004). As shown in Eq. 3, the probability of selecting a certain rank order depends on all the latent traits assessed in the block.

### Independence Likelihood

The original implementation of the Thurstonian IRT model (Brown & Maydeu-Olivares 2011, 2012) is based on the binary outcomes of all pairwise item comparisons within a block. For example, in a block of size $B=3$, there are $B(B-1) / 2 = 3$ pairwise comparisons, between items 1 and 2, 1 and 3, and 2 and 3. In this way, each rank order can be equivalently written as a set of binary (0,1) outcomes coding which item in the pair was preferred. For example, the rank order 2–1–3 would be recoded into the binary outcomes 0–1–1 for the pairwise comparisons between items 1 and 2, 1 and 3, and 2 and 3. Assuming items *i* and *l* measure traits 1 and 2, respectively, the probability that item *i* is preferred over item *l* is a normal ogive function:

4 $$\begin{aligned} P(Y_{jil} = 1 | \varvec{\theta }_{j}) = \Phi \left( \frac{- \gamma _{il} + \lambda _i\theta _{j1} - \lambda _l\theta _{j2}}{\sqrt{\psi _i^2 + \psi _l^2}}\right) \end{aligned}$$

where $\Phi (x)$ denotes the cumulative standard normal distribution function evaluated at *x* and $\gamma _{il}$ denotes the intercept for the pairwise comparison.

Let $S_k$ denote the set of item indices belonging to block *k*. Let *o* denote the observed binary outcome with $o \in \{0,1\}$ and $P(Y_{jil} = 0) = 1 - P(Y_{jil} = 1)$. Under the assumption of local independence of the binary outcomes within each block, the probability of selecting rank order *r* is:

5 $$\begin{aligned} P^{\text {Independence}}(X_{jk}=r | \varvec{\theta }_{j}) = \prod _{i,l \in S_k; i < l}P(Y_{jil} = o | \varvec{\theta }_{j}) \end{aligned}$$

The assumption of local independence is incorrect for block size $B > 2$. Under the original implementation, the item parameters and trait correlations are estimated via least squares so that the estimation does not rely on the (possibly) incorrect likelihood.

Note that with item parameter estimates from the standard Thurstonian IRT implementation, Eq. 3 does not yield a correct probability measure. That is, across the *R* possible rank orders, the probabilities do not add up to one ($\sum _{r=1}^R P(X_{jk}=r | \varvec{\theta }_{j}) \ne 1$). This is because the restriction on the pairwise comparison intercepts $\gamma _{il} = \mu _i - \mu _l$ is not imposed. Therefore, to work with Eq. 3, the standard Thurstonian IRT implementation has to be slightly modified. For example, for block size $B = 3$ the set of restrictions on the intercepts of the block containing items 1,2, and 3 is:

6 $$\begin{aligned} \gamma _{12}&= \mu _1 - \mu _2 \nonumber \\ \gamma _{13}&= \mu _1 - \mu _3 \nonumber \\ \gamma _{23}&= \mu _2 - \mu _3 \end{aligned}$$

Because of linear dependencies, the set of equations in 6 can be reduced, in the case of $B=3$, to one equation involving all three intercepts, for example, $\gamma _{12} = \gamma _{13} - \gamma _{23}$^2^.

### Block and Test Information

#### Block Information Based on the Genuine Likelihood

The information for a block and a single rank order *r* is the negative of the Hessian of the logarithmized response probability for the latent traits, where *H*(*f*) denotes the Hessian of function *f*.

7 $$\begin{aligned} {\textbf{I}}_{jkr} = \mathbf {-H}\left( \log P(X_{jk}=r)\right) \end{aligned}$$

Obtaining the Hessian for a multidimensional response probability involves differentiating twice for each pair of traits in both orders. Hence, for *F* latent traits, ${\textbf{I}}_{jkr}$ is an $F \times F$ matrix. For example, to obtain the entry in the second row and first column, ${\textbf{I}}_{jkr}(1,2)$, the response probability $P(X_{jk}=r)$ is first differentiated for Trait 1 and then for Trait 2. As for the response probability, there is no analytical solution for the Hessian, but numerical approximation is feasible. Here, the implementation in the R function optim() with the argument hessian set to TRUE was used. Fisher information—or likewise expected block information ${\textbf{I}}_{jk}$—is calculated as the expectation across all $R=B!$ possible rank orders:

8 $$\begin{aligned} {\textbf{I}}_{jk} = \sum _{r=1}^{R} {\textbf{I}}_{jkr} P(X_{jk}=r) \end{aligned}$$

In the following, the term block information is used for expected block information if not explicitly indicated otherwise.

#### Block Information Based on the Independence Likelihood

For information based on the independence likelihood, the analytical formula can be given. Assuming items *i* and *l* measure the traits indexed with 1 and 2, respectively, the information for the observed binary outcome *o* is given by:

9 $$\begin{aligned} {\textbf{I}}_{jilo} = \left( \log (P(Y_{jil} = o))\right) '' = \frac{P''(Y_{jil} = o)}{P(Y_{jil} = o)} - \left( \frac{P'(Y_{jil} = o)}{P(Y_{jil} = o)}\right) ^2 \end{aligned}$$

with $P'(Y_{jil} = 1)$ being the first derivate of $P(Y_{jil} = 1)$ given in Eq. 4:

10 $$\begin{aligned} P'(Y_{jil} = 1) = \frac{1}{\sqrt{\psi _i^2 + \psi _l^2}} \begin{pmatrix} \lambda _i \\ -\lambda _l \end{pmatrix}' \phi \left( \frac{- \gamma _{il} + \lambda _i\theta _{j1} - \lambda _l\theta _{j2}}{\sqrt{\psi _i^2 + \psi _l^2}}\right) \end{aligned}$$

where $\phi (x)$ denotes the standard normal probability density function evaluated at *x*, $P'(Y_{jil} = 0) = - P'(Y_{jil} = 1)$, and $P''(Y_{jil} = 1)$ denotes the second derivate of $P(Y_{jil} = 1)$:

11 $$\begin{aligned} P''(Y_{jil} = 1) = \begin{pmatrix} \frac{\gamma _{il} - \lambda _i\theta _j + \lambda _l\theta _j}{\sqrt{\psi _i^2 + \psi _l^2}} \\ \frac{\gamma _{il} - \lambda _i\theta _j + \lambda _l\theta _j}{\sqrt{\psi _i^2 + \psi _l^2}} \end{pmatrix} P'(Y_{jil} = 1) \end{aligned}$$

Fisher information—or likewise the expected information based on the independence likelihood ${\textbf{I}}_{jil}$—is calculated as the expectation over the possible outcomes $o \in \{0,1\}$ (Brown & Maydeu-Olivares 2018b):

12 $$\begin{aligned} {\textbf{I}}_{jil}&= \sum _{o \in \{0,1\}}{\textbf{I}}_{jilo}P(Y_{jil} = o) \nonumber \\&= \frac{1}{\psi _i^2 + \psi _l^2} \begin{pmatrix} \lambda _i^2 &{} -\lambda _i\lambda _l &{} \cdots &{} 0 \\ -\lambda _i\lambda _l &{} \lambda _l^2 &{} \cdots &{} 0 \\ \vdots &{} \vdots &{} \ddots &{} \vdots \\ 0 &{} 0 &{} \cdots &{} 0 \\ \end{pmatrix} \frac{P'(Y_{jil} = 1)^2}{P(Y_{jil} = 1)\left( 1 - P(Y_{jil} = 1)\right) } \end{aligned}$$

Block information based on the independence likelihood is obtained by summing over all pairwise item comparisons in a block. Let $S_k$ denote the set of item indices belonging to block *k*.

13 $$\begin{aligned} {\textbf{I}}_{jk}^{\text {Independence}} = \sum _{i,l \in S_k; i < l}{\textbf{I}}_{jil}^\star \end{aligned}$$

where ${\textbf{I}}_{jil}^\star$ can denote either observed information ${\textbf{I}}_{jilo}$ (Eq. 9) or Fisher information ${\textbf{I}}_{jil}$ (Eq. 12). Note that the pairwise outcomes do not contribute independent information when block size $B>2$. Specifically, of the *B*! pairwise comparisons in each block, $B(B-1)(B-2)/6$ are redundant (Brown & Maydeu-Olivares 2011). Thus, information based on the independence likelihood is higher than that based on the genuine likelihood.

#### Test Information

When each item is presented in only one block, as is typically done in MFC tests, test information is obtained by summing block information across all blocks in the test:

14 $$\begin{aligned} {\textbf{I}}_{jT} = \sum _{k=1}^{K} {\textbf{I}}_{jk}^\star \end{aligned}$$

Here, ${\textbf{I}}_{jk}^\star$ can denote the four block information estimators described above: For the genuine likelihood, the observed information ${\textbf{I}}_{jkr}$ or the Fisher information ${\textbf{I}}_{jk}$, and for the independence likelihood, ${\textbf{I}}_{jk}^{\text {Independence}}$ based on observed information ${\textbf{I}}_{jilo}$ or on Fisher information ${\textbf{I}}_{jil}$.

Posterior information is obtained by adding prior information for the latent traits, for example, for a multivariate normal prior with covariance matrix $\mathbf {\Sigma }$, ${\textbf{I}}_{jT}^\text {posterior} = {\textbf{I}}_{jT} + \mathbf {\Sigma }^{-1}$. Then, the estimation variances for a trait vector are obtained as the diagonal of the inverse of expected or observed test information:

15 $$\begin{aligned} \mathbf {\sigma }^2_{jT} = {{\,\textrm{diag}\,}}\left( {\textbf{I}}_{jT}^{-1}\right) \end{aligned}$$

Standard errors are obtained by then taking the square root:

16 $$\begin{aligned} \mathbf {\sigma }_{jT} = \sqrt{\mathbf {\sigma }^2_{jT}} \end{aligned}$$

The IRT-based computation of *SE*s typically uses Fisher information for ${\textbf{I}}_{jT}$. However, in standard software programs, the *SE*s are derived numerically by default. That is, they are based on the negative Hessian of the log-likelihood at the trait estimate. This is equivalent to substituting observed test information for $I_{jT}$. For block size $B>2$, the standard errors based on the independence likelihood are smaller than those based on the genuine likelihood (Yousfi 2020). That is, they have a negative bias. Based on their simulations, Brown & Maydeu-Olivares (2011) judge that the resulting overestimation of reliability is negligible.

## Simulation Study 1: Simulation on Standard Error Accuracy

In this simulation, I investigate the accuracy of *SE*s based on different formulations of Thurstonian IRT block and test information. Specifically, first, I investigate whether *SE* accuracy is affected by using the independence likelihood (Eq. 4) instead of the genuine likelihood (Eq. 3; RQ1). The difference between the two formulations is that block information can account for local dependencies that occur for block sizes $B>2$. Thus, there should be no difference in accuracy between the two formulations for block size $B=2$. For block sizes $B>2$, the difference should increase with the block size. Second, I compare the accuracy of expected versus observed *SE*s (RQ2). Since obtaining expected *SE*s under the Thurstonian IRT model and the genuine likelihood is computationally intensive, it is worth investigating whether the computationally cheaper observed *SE*s are comparable in terms of precision.

The accuracy of the *SE*s was examined under various test design conditions that influence the amount of information and for two types of estimators—maximum likelihood (ML) and maximum a posteriori (MAP). The MAP estimator is most often used for Thurstonian IRT models (e.g., Brown & Maydeu-Olivares 2011; Wetzel & Frick 2020).

### Method

MFC responses were simulated for five traits, a test with a block size of three and half of the pairwise item comparisons across the test involving items keyed in different directions (i.e., one positive, one negative factor loading). Item keying was chosen so that the accuracy of the *SE*s would not be confounded with ipsativity. Ipsativity with all positively keyed items was observed in simulations (e.g., Bürkner et al. 2019; Frick et al. 2023). The design matrix showing which items loaded on which traits can be found on GitHub: https://github.com/susanne-frick/MFCblockInfo. Item intercepts $\mu _i$ were drawn from $U(-1,1)$. Item uniquenesses $\psi _i^2$ were calculated as $1-\lambda _i^2$ (i.e., standardized item utilities were simulated). Errors were drawn from $N(0, \psi _i^2)$. For the second trait, the trait levels varied from $-2$ to $+2$ in steps of 0.5. The other traits were fixed to 0. This yielded nine trait vectors. Traits were estimated with box constraints to be within the range of $[-3, 3]$. Otherwise unreasonably large estimates were obtained in some cases. This is because precision is typically lower for extreme trait values. Note that box constraints are typically imposed for estimating traits in IRT models. For example, under the two-parameter logistic model, the ML estimate for a response vector with all zeros or ones is infinite. The MAP estimator can alleviate this issue by pulling estimates toward the mean.

Six factors were varied and completely crossed: first, the *likelihood* used for estimating the traits and their *SE*s was either the genuine likelihood or the independence likelihood. Second, the *type of SE*s: Both observed and expected *SE*s were computed. Besides these two factors that were of main interest for the research questions, the trait estimator and the test design were varied: Third, the *type of estimator* was either ML or MAP. For the MAP estimator, a multivariate normal prior with a mean vector of zero and correlations based on meta-analytic correlations between the Big Five (van der Linden et al. 2010) were used. The correlations are shown in Table 1. Fourth, the *size of the factor loadings*: High factor loadings were drawn from *U*(.65, .95) and low factor loadings were drawn from *U*(.45, .75). Fifth, the *test length* was either short (60 pairwise comparisons) or long (120 pairwise comparisons). Sixth, the *block size* was either two, three, or four. The number of pairwise comparisons, and with it the approximate amount of information, was kept constant across block sizes. This constancy implies that the number of items varied. Thus, for block sizes two, three, and four, the short version comprised 60, 20, and 10 blocks, made out of 120, 60, and 40 items, respectively. For the long version, the test design was duplicated. In this setting, information decreases with increasing block size due to the local dependencies. In contrast, information would increase with block size if the amount of items was kept constant.

**Table 1:** Correlations used in the simulation studies.

Trait	E	O	A	C
N	$-.36$	$-.17$	$-.36$	$-.43$
E		.43	.26	.29
O			.21	.20
A				.43

N = neuroticism, E = extraversion, O = openness, A = agreeableness, C = conscientiousness. These are meta-analytic correlations between the Big Five as reported by van der Linden et al. (2010).

To operationalize *SE* accuracy, I examined the extent to which the *SE* estimates correspond with empirical *SE*s. The empirical *SE*s were defined as the standard deviation of trait estimates across *M* responses based on the same trait vector *j* to test *q* (cf., Ippel & Magis 2020; Paek & Cai 2014):

17 $$\begin{aligned} {{\,\textrm{SE}\,}}_q \left( \theta _j\right) = \frac{\sum _{m=1}^{M} \left( \hat{\theta }_{jm} - \bar{\hat{\theta }}_{j}\right) ^2}{M-1} \end{aligned}$$

Hence, for each unique trait vector *j* and each test *q*, *M* response vectors are simulated. The traits $\theta _{jm}$ are then estimated for each of the $m = 1 \dots M$ response vectors. Empirical *SE*s are the standard deviation of these *M* trait estimates.

All *SE*s are computed at the true trait value to not confound *SE* accuracy with the accuracy of the trait estimate. However, since in practice *SE*s can only be computed at the trait estimate, the results for this case can be found in the appendix. The four types of *SE*s (expected vs. observed $\times$ genuine vs. independence likelihood) are obtained by substituting the corresponding block information estimator into the equation for test information (Eq. 14) and then computing the *SE*s based on this test information estimate (Eq. 16).

#### Simulation Procedure

All data generation and analysis were carried out in R (R Core Team 2020), using the R packages doMPI (Weston 2017), mvtnorm (Genz et al. 2020), numDeriv (Gilbert & Varadhan 2019), psych (Revelle 2019), gridExtra (Auguie 2017), and ggplot2 (Wickham 2016). For each combination of test design, estimator, likelihood, and trait level, 200 tests were simulated, yielding a total of $2 \times 2 \times 2 \times 3 \times 2 \times 9 \times 200 = 86,400$ tests. For each test, item parameters were drawn according to the test design, and $M=500$ response vectors were simulated. Traits were estimated for each response vector, either based on the genuine or on the independence likelihood, depending on the condition. Then, the two types of *SE*s (expected and observed) were computed at the trait estimate. That is, both types of *SE*s were computed in each condition. Trait recovery and the accuracy of the *SE*s for the second trait were assessed by computing the mean bias (*MB*) and root mean square error (*RMSE*) with the following formulas, where $\xi$ denotes the true parameter and $\hat{\xi }_m$ its estimate for response *m*:

18 $$\begin{aligned} MB(\xi )&= \frac{\sum _{m=1}^{M}\left( \hat{\xi }_m - \xi \right) }{M} \end{aligned}$$

19 $$\begin{aligned} RMSE(\xi )&= \sqrt{\frac{\sum _{m=1}^{M}\left( \hat{\xi }_m - \xi \right) ^2}{M}} \end{aligned}$$

The MB and RMSE were computed for the latent traits $\theta$ and their observed *SE*s. In addition, for the *SE*s, the mean ratio (*MR*) between estimated and true *SE*s was computed to get a sense of the proportional size of over- or underestimation:

20 $$\begin{aligned} MR(\xi ) = \frac{\sum _{m=1}^{M} \hat{\xi }_m}{M\xi } \end{aligned}$$

For the *SE*s, the empirical *SE* computed with Eq. 17 served as the true parameter. The expected *SE*s do not differ across the *M* response vectors. Thus, for the expected *SE*s, *MB* and *RMSE* are equal and simplify to the bias: $\hat{\xi } - \xi$. Similarly, the MR simplifies to a ratio: $\hat{\xi } / \xi$. The MB and RMSE of the *SE*s were summarized with means and *SD*s by condition, and the amount of variance explained by the contrasts between the conditions was calculated in an ANOVA framework.

### Results

In reporting the results, in line with RQs 1 and 2, I focus on the bias of the *SE*s depending on the type of likelihood and the type of *SE*s and on their interaction with test design factors and the type of estimator. The results for the bias of the trait estimates and the size of empirical *SE*s are reported in supplementary online material.

**Table 2:** Variance in bias for information-based standard errors explained in % by the manipulated factors in simulation study 1 on standard error accuracy.

Factor	MB	MR	RMSE
Blocksize	5	4	7
Estimator	37	35	–
Estimator$\times$blocksize	1	0	4
Estimator$\times$length	5	3	–
Estimator$\times$likelihood	0	0	7
Estimator$\times$likelihood$\times$blocksize	0	0	4
Length	0	1	14
Likelihood	9	8	1
Likelihood$\times$blocksize	6	5	1
Loadings	0	0	2
Loadings$\times$estimator	1	0	0
Loadings$\times$estimator$\times$likelihood	0	0	1
Loadings$\times$estimator$\times$likelihood$\times$blocksize	0	0	1
Loadings$\times$likelihood	1	0	–
Loadings$\times$likelihood$\times$blocksize	1	0	–
Residuals	34	43	55

MB = mean bias, MR = mean ratio, RMSE = root mean squared error. For the expected *SE*s, the bias and the ratio are shown. The RMSE was only computed for observed *SE*s.

**Table 3:** Means of bias for information-based standard errors by condition in simulation study 1 on standard error accuracy.

Method	Blocksize	Likelihood	Estimator	MB		MR		RMSE	
Expected	2	Genuine	ML	$-0.02$	(0.03)	0.94	(0.05)		
			MAP	0.05	(0.05)	1.16	(0.15)		
		Independence	ML	$-0.02$	(0.03)	0.94	(0.06)		
			MAP	0.05	(0.05)	1.16	(0.15)		
	3	Genuine	ML	$-0.03$	(0.04)	0.94	(0.06)		
			MAP	0.06	(0.05)	1.18	(0.18)		
		Independence	ML	−0.07	(0.04)	0.84	(0.06)		
			MAP	0.01	(0.05)	1.04	(0.16)		
	4	Genuine	ML	$-0.04$	(0.05)	0.93	(0.08)		
			MAP	0.07	(0.06)	1.20	(0.20)		
		Independence	ML	$-0.12$	(0.06)	0.77	(0.07)		
			MAP	$-0.02$	(0.05)	0.95	(0.18)		
Observed	2	Genuine	ML	$-0.02$	(0.03)	0.95	(0.05)	0.03	(0.02)
			MAP	0.05	(0.05)	1.16	(0.16)	0.05	(0.04)
		Independence	ML	$-0.02$	(0.03)	0.95	(0.06)	0.03	(0.02)
			MAP	0.05	(0.05)	1.16	(0.15)	0.05	(0.04)
	3	Genuine	ML	$-0.03$	(0.04)	0.94	(0.06)	0.04	(0.03)
			MAP	0.06	(0.05)	1.18	(0.18)	0.06	(0.05)
		Independence	ML	$-0.07$	(0.04)	0.85	(0.06)	0.07	(0.04)
			MAP	0.01	(0.05)	1.04	(0.16)	0.04	(0.04)
	4	Genuine	ML	$-0.03$	(0.05)	0.94	(0.08)	0.05	(0.03)
			MAP	0.07	(0.06)	1.20	(0.20)	0.07	(0.06)
		Independence	ML	$-0.12$	(0.06)	0.77	(0.07)	0.12	(0.05)
			MAP	$-0.02$	(0.06)	0.96	(0.18)	0.05	(0.03)

MB = mean bias, MR = mean ratio, RMSE = root mean squared error, ML = maximum likelihood, MAP = maximum a posteriori. Standard deviations are given in parentheses.

#### RQ1: Bias of *SE*s Based on the Genuine Versus the Independence Likelihood

For the MB and the MR, the type of likelihood interacted with the block size (Table 2). Interactions with the other factors (estimator, test length and size of loadings) were negligible. For the genuine likelihood, the *SE* estimates had a small positive bias (mean MB = 0.02, Table 3). For the independence likelihood, as expected, the *SE* estimates had a negative bias for block sizes $B>2$ and this bias increased with block size (mean MB = $-$0.03 and $-$0.07 for block sizes 3 and 4, respectively). This bias was smaller for the MAP estimator (mean MB = 0.01 and $-$0.02 for block sizes 3 and 4, respectively). This was probably because the positive bias in the estimator and the negative bias in the likelihood counteract.

**Fig. 2:**
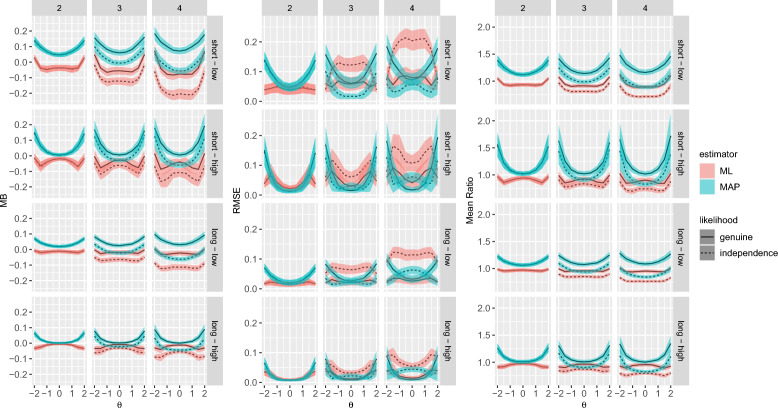
Bias for the observed standard errors in Simulation Study 1 on standard error accuracy. Shaded areas show $\pm 1$ *SD* around the mean (line). MB = mean bias, RMSE = root mean square error, ML = maximum likelihood, MAP = maximum a posteriori.

**Fig. 3:**
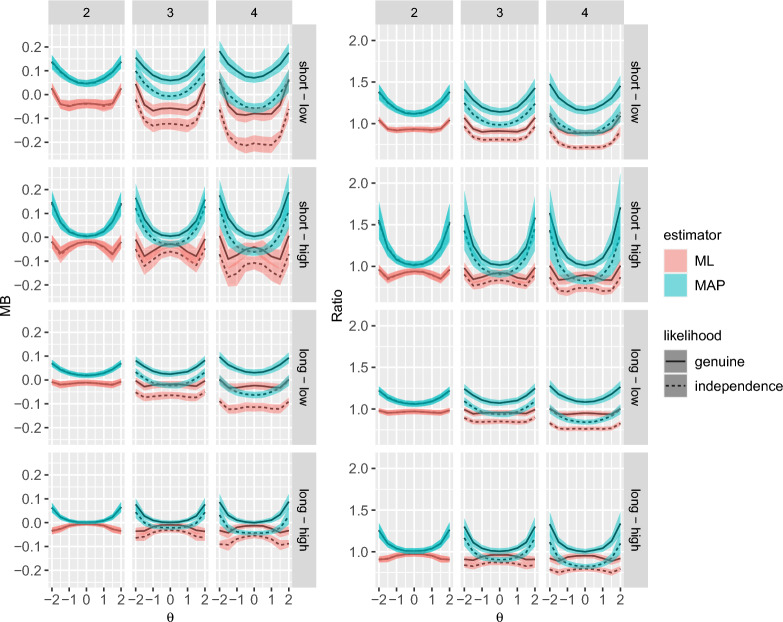
Bias for the expected standard errors in Simulation Study 1 on standard error accuracy. The top row shows results for the short test (20 blocks) and the bottom row shows results for the long test (40 blocks). Shaded areas show $\pm 1 SD$ around the mean (line). MB = mean bias, ML = maximum likelihood, MAP = maximum a posteriori.

To quantify the size of the bias, the ratio between true and estimated *SE*s (MR) was computed. For mean trait levels ($\theta = 0$), the MR was acceptable and ranged between 0.76 and 1.31 for the genuine likelihood and between 0.63 and 1.21 for the independence likelihood (Figs. 2 and 3). With the MAP estimator, it was unacceptably high for extreme trait levels ($\theta = \pm 2$) with maxima of 3.88 and 3.46 for the genuine and the independence likelihood, respectively.

For the RMSE, the type of likelihood interacted with the type of estimator and with the block size. Overall, the effects were similar to the MB and MR. The ML estimator with the independence likelihood had the largest RMSE (mean RMSE = 0.08). Remarkably, this was even higher for medium than for extreme trait levels (Fig. 2) which might be attributable to the box constraints.

#### RQ2: Bias of Observed Versus Expected *SE*s

The MB of the *SE* estimates was generally low (mean = $-$0.01, *SD* = 0.07, Table 3). The difference in the MB of the observed and expected *SE*s was negligible, explaining 0% of the variance across trait levels (Table 2, Figs. 2 and 3). The same results were found for the MR.

### Discussion

Regarding the comparison between the genuine and the independence likelihood, especially when information was low (i.e., because the loadings were low or the test was short), the independence likelihood for block size $B>2$ resulted in a bias of the *SE* estimates that was not negligible in relation to the scale of the traits and their empirical *SE*s. Interestingly, for the MAP estimator, the independence likelihood resulted in a smaller bias, because the negative bias in the likelihood probably counteracted the positive bias in the estimator.

Regarding the comparison between observed and expected *SE*s, the results showed that the observed *SE*s were as accurate as the expected ones. Thus, when only the test level information is of interest, researchers can rely on the observed information at the trait estimate, thus saving computational time and resources.

In this simulation, I focused on test design factors that are relevant for the level of information, keeping other design factors constant, such as the number of traits, the trait correlations, and the number of comparisons between mixed keyed items. Future studies varying these test design factors might yield more pronounced differences between the types of the *SE*s and of the likelihood.

## Assembling MFC Tests Based on Block Information

Standard errors are only partially informative about the accuracy of block information because their computation involves summing across blocks. The second part focuses on whether block information is sufficiently accurate to be used in test construction. Since block information is multidimensional, the first step is to summarize it into a scalar. Therefore, in the following section, I first present possibilities to summarize the multidimensional block information into one scalar or a scalar for each trait, called *information summaries*. The second step is to simulate the test construction process based on block information. Since manual test construction cannot be simulated, automated test assembly (ATA) is simulated instead. Before, I give some details on ATA algorithms and how they can be combined with block information summaries.

### Information Summaries

#### Information Summaries from Optimal Design

Several information summaries originate from the optimal design literature and have been used in multidimensional computerized adaptive testing (CAT) and sometimes in multidimensional ATA (Debeer et al. 2020). In MFC tests, the investigator is usually interested in all the traits. Therefore, I focus on the summaries that weigh all the traits equally.

Out of them, the sum of the sampling variances and the determinant of the information matrix performed best in an MFC CAT simulation (Lin 2020). Minimizing the sum of the sampling variances across the test (Eq. 15), based on expected test information, is called A-optimality. Maximizing the determinant of the test information matrix (Eq. 14) is called D-optimality. Hence, optimizing the sum of the sampling variances or the determinant depends on the information matrix being non-singular. In most cases, the information matrix for a single block is not invertible because the latent trait space is identified only when there are several blocks and no linear dependencies between factor loadings $\lambda$, that is, when the pairwise comparison matrix of factor loadings $\mathbf {\Lambda }$ has full rank (for details, see Brown 2016). In the special case in which each block measures all *F* traits, the information matrix may be invertible. Therefore, for MFC tests, the sum of the sampling variances or the determinant can usually only be optimized for several blocks at once (i.e., for test information). Alternatively, non-singularity can be achieved by adding a prior for the distribution of the latent traits to the block information matrix.

By contrast, maximizing the trace of the information matrix, called T-optimality, does not depend on a positive-definite matrix (Eq. 8). However, it ignores the impact of trait correlations (Lin 2020). Maximizing T-optimality performed worst in an MFC CAT simulation (Lin 2020). However, it has the advantages that it is additive across blocks and that it can be calculated for a single block without a prior.

#### Block ${R^2}$

There are some situations, where the information summaries from the optimal design literature cannot be used or might at least show suboptimal properties. First, a test constructor might want to assess the blocks of a fixed test instead of constructing a new one. Second, some test construction steps might be difficult to formalize into a test assembly problem. Third, some test constructors might prefer to visually inspect block properties in conjunction with the item content.

In these situations, the full information matrix is difficult to interpret. The posterior sampling variances (or their sum) could be used, but for a single block, the prior might be too influential (see the following simulation studies). The same applies to the determinant of the posterior block information matrix. The diagonal entries of the block information (or their sum) have the disadvantage to ignore the contribution from correlated traits.

Therefore, for manually inspecting the blocks of a fixed test, I propose a new information summary, which I will call block $R^2$. Block $R^2$ quantifies the proportional reduction in the sampling variances of the traits that is achieved by including this block. To compute block $R^2$, first, test information ${\textbf{I}}_{jT}$ (Eq. 14) based on the Fisher information (Eq. 8) must be calculated for two sets of blocks: for a set *T*, which includes the respective block *k*, and for a set $T \setminus k$, which excludes it. Second, sampling variances are calculated for both sets by applying Eq. 15. Third, block $R^2$ is obtained such that higher values indicate a larger reduction in the sampling variances:

21 $$\begin{aligned} {\textbf{R}}_{jk}^2 = 1 - \frac{\varvec{\sigma }^2_{jT}}{\varvec{\sigma }^2_{jT \setminus k}} \end{aligned}$$

Thus, for *F* latent traits, block $R^2$ is a vector of length *F*. It follows from this procedure that block $R^2$ is relative to the set *T* of reference blocks. However, this also applies to the item parameters in general since their estimation depends on the whole test and the sample. In most practical applications, the set of reference blocks will be all blocks that are being assessed. Alternatively, it can be a subset of blocks that form a test that should be extended.

### Automated Test Assembly

In this manuscript, automated test assembly (ATA) first serves as a vehicle to simulate the test construction process. However, beyond that, it might be particularly promising for MFC tests. This is because constructing MFC tests can be a combinatorial challenge, because it might involve not only information maximization, but also the balancing of item keying and the numbers of items per trait as well as social desirability matching (e.g., Brown & Maydeu-Olivares 2011; Wetzel & Frick 2020). Please note that in here the focus is on selecting blocks from fixed item compositions, not on assembling new blocks from possible item comparisons. The latter bears the danger to elicit unknown item interactions.

In ATA, items are selected from a pool so that a criterion is maximized (or minimized) and certain restrictions are fulfilled (van der Linden 2005). For example, information is maximized while holding the number of items per trait equal. Practical applications of ATA include constructing parallel test forms with similar information curves or a test with peaked information at a certain trait level for selection purposes. For example, employers might be interested in selecting all applicants who score two standard deviations above the mean. By contrast, in CAT, a unique test is assembled for each individual respondent so that information is maximized at her/his trait level. For an introduction to ATA, see van der Linden (2005).

**Table 4:** Miniature example for an automated test assembly problem.

k	${\varvec{s}}(\varvec{\theta }_1)$	${\varvec{s}}(\varvec{\theta }_2)$	${\varvec{C}}_{\cdot 1}$	${\varvec{C}}_{\cdot 2}$	${\varvec{x}}$
1	5	3	1	1	1
2	3	1	1	0	0
3	2	3	1	1	1
4	6	2	1	0	1
5	4	1	1	1	0
$\sum _{K}$	20	10	5	3	
$\sum _{k \in x}$	12	5	3	2	

Solution for $d_1 = 3$, $d_2 = 2$ and weights $w_1 = 1$, $w_2 = 0.5$.

#### Mixed Integer Programming With a Maximin Criterion

Mixed integer programming (MIP) algorithms are the first choice for ATA because they can find the optimal solution if it exists. Moreover, they can incorporate a *maximin* criterion, which has good properties and is particularly suited to IRT (van der Linden 2005). In IRT, information varies across trait levels. Only a single value can be maximized, that is, information at one trait level. Here, the maximin criterion comes into play: Information at a reference trait level is maximized, while constraints keep the (relative) distance to a test information curve minimal. When the information at the reference trait level increases, the information at all other trait levels increases proportionally. In this way, the test information curve can have a specified shape and be maximized at the same time. The desired shape of test information is often called a target information curve. An alternative approach is a weighted criterion. Here, a weighted average of information across trait levels is maximized. This has the disadvantages that low information for some trait levels can be compensated by high information for others and the shape of the test information curve cannot be controlled. In order to apply MIP, a test assembly problem has to be framed as a (constrained) linear optimization problem. Next, I describe how assembling an MFC test from a block pool can be framed for MIP with a block information summary as a relative maximin criterion. To better illustrate the procedure, a toy example with five blocks, two grid points, and two constraints is given in Table 4.

First, $g = 1,\dots , G$ trait levels are defined for which information is to be computed. In the multidimensional case, typically, a grid of trait levels is selected, for example, all combinations of $-1$, 0, and 1 across five traits (e.g., Debeer et al. 2020; Veldkamp 2002). In the example (Table 4), two grid points are defined. Then, for each grid point vector $\varvec{\theta }_g$ and each block *k*, a scalar information summary $s_k(\varvec{\theta }_g)$ is calculated, for example, the trace of the test information matrix. In Table 4, the information summaries for each block are displayed in the columns labeled ${\varvec{s}}(\varvec{\theta }_1)$ and ${\varvec{s}}(\varvec{\theta }_2)$.

Whether block *k* is included in the test is encoded in a decision vector ${\textbf{x}} = (x_1,\dots ,x_K)'$, taking on a value of 1 if the block is included and 0 otherwise. Then, the task is to find the values of ${\textbf{x}}$ for which the summary $y = \sum _{k=1}^{K}s_k(\varvec{\theta }_1)x_k$ at an arbitrary reference point vector $\varvec{\theta }_1$ is maximized. That is, *y* is the sum of the information summary $s_k$ for all blocks included in the test at the reference point vector $\varvec{\theta }_1$.

To obtain a relative criterion, first, weights are computed for each grid point vector $\varvec{\theta }_g$: The information summary *s* is summed across all *K* blocks and weighted by this sum for the reference point vector $\varvec{\theta }_1$ to obtain a weight $w_g$:

22 $$\begin{aligned} w_g = \frac{\sum _{k=1}^{K}s_k(\varvec{\theta }_g)}{\sum _{k=1}^{K}s_k(\varvec{\theta }_1)} \end{aligned}$$

In the toy example given in Table 4, the sums of the information summaries across all blocks are $\sum _{k=1}^{K}s_k(\varvec{\theta }_1) = 20$ and $\sum _{k=1}^{K}s_k(\varvec{\theta }_2) = 10$. Setting $\varvec{\theta }_1$ as the reference point vector (i.e., $w_1 = 1$) results in a weight of $w_2 = 10/20 = 0.5$ for the second grid point vector.

Next, the maximin criterion can be formulated: Maximize the information summary at the reference point vector $\varvec{\theta }_1$, while constraints ensure that the summary at the other points is close to proportional to their value in the block pool:

23 $$\begin{aligned} \text {maximize } y \end{aligned}$$

subject to

24 $$\begin{aligned} \sum _{k=1}^{K}\left( s_k(\theta _g) x_k \right) - w_g y \ge 0 \quad \text {for all} \quad g \end{aligned}$$

In the example, the criterion value of the solution, which is the sum of the information summary across the selected blocks for $\varvec{\theta }_1$, is $y = 12$. Then, for $\varvec{\theta }_2$ the sum is $5 \le 0.5*12$.

Additional constraints can be added to the ATA problem. The blocks’ values on the $n = 1, \dots , N$ constrained attributes are encoded in a $K \times N$ matrix ${\textbf{C}}$, and the minimum,^3^ values for the constraints are encoded in a vector ${\textbf{d}} = (d_1, \dots d_N)'$.

25 $$\begin{aligned} \sum _{k=1}^{K}c_{kn} x_k \ge d_n \quad \text {for all} \quad n \end{aligned}$$

In the example, the first constraint encoded in column ${\textbf{C}}_{\cdot 1}$ is test length. The value on this constraint is 1 for each block. The final test length should be three, that is, $d_1=3$. The second constraint could be that the test should include at least 2 blocks measuring Trait 1, that is, $d_2=2$. This is encoded in the second column ${\textbf{C}}_{\cdot 2}$ that takes on a value of 1 for all blocks measuring Trait 1 and 0 otherwise. In the final solution, there are three blocks (1, 3, and 4) out of which two measure Trait 1 (1 and 3).

MIP methods are applicable only to information summaries that are linear across items (or blocks). In the multidimensional case, linear approximations to item information can be used (e.g., Debeer et al. 2020; Veldkamp 2002), but linear approximation is not possible with MFC block information because there is no closed-form expression for it. Of the information summaries derived from the optimal design literature, only the trace of the information matrix can be used to construct MFC tests with MIP because the trace is the only one that is additive (and correspondingly linear) across blocks.

#### Heuristics

Because the trace performed worst in MFC CAT simulations (Lin 2020), I also investigated ATA algorithms that can be used with the sum of the sampling variances and the determinant, both of which performed well in previous simulations (Brown 2012; Lin 2020; Mulder & van der Linden 2009). These algorithms are heuristics that can be combined with all the criteria described above. In contrast to MIP methods, heuristics are guaranteed to find a solution, but the solution is not guaranteed to be optimal (van der Linden 2005).

The simplest heuristics are constructive heuristics, which sequentially select a locally optimal item (or block). For example, Veldkamp (2002) compared the performance of a greedy heuristic for ATA with multidimensional items to that of MIP (with a linear approximation of item information). More sophisticated heuristics are local search heuristics that introduce randomness into the selection process to prevent the search from being trapped in a suboptimal space, often inspired by natural processes. For example, Olaru et al. (2015) compared, among others, a genetic algorithm and ant colony optimization for the assembly of a short scale. However, local search heuristics are more specifically tailored to a certain problem than MIP.

## Simulation Study on Test Construction

In this simulation, I compare the performance of different information summaries for test construction (RQ3). Expected information is computed based on the genuine likelihood. Both the genuine likelihood and expected information potentially provide more accurate information. This might matter on the block level where precision is lower than on the test level. In addition, using expected information is consistent with the typical definition of item information in IRT and its use in test assembly algorithms. The simulation is designed to obtain a first impression of the performance of the criteria and algorithms in a simple setting. Therefore, the composition of the block pool was ideal with respect to the balancing of traits and item keying. That is, all possible combinations of traits to blocks occurred equally often and half of the pairwise comparisons were between differently keyed items. Simulations with all the items keyed equally are given in supplementary online material. I investigate three different targets. The first two focus on the assembly of a test for a general population. The third focuses on the assembly of a test that is to be used as a screening instrument with highest information at a cut-off point. Moreover, posterior information is computed. This is because with posterior information, the information matrix is invertible even for a small number of blocks which is necessary for optimizing the variances and the determinant. In preliminary simulations, the results for an ML estimator did not differ qualitatively.

Given that local search heuristics are specifically tailored to certain problems, in this simulation, I use a simple greedy heuristic instead. Developing a sophisticated greedy algorithm or local search heuristic is beyond the scope of this manuscript (for examples of such algorithms, see, Kreitchmann et al. 2021; Luecht 1998; Olaru et al. 2015). This greedy heuristic sequentially selects the block with the smallest variances or highest determinant, respectively, weighted across trait levels. The results can serve as a benchmark of what might be achieved with these information summaries and a more elaborate local search heuristic.

The performance of the information summaries from the optimal design literature in conjunction with ATA algorithms is compared with that of mean block $R^2$, mean posterior variances (calculated for each block separately), mean absolute loadings within blocks, and random block selection. The mean of the absolute loadings within blocks serves as an approximation of the practice of selecting items based (primarily) on the size of their loadings. Block $R^2$ is calculated by using the whole block pool as the reference set *T*, which makes block $R^2$ independent of the previously selected items. In this setting, the optimal solution for mean block $R^2$, mean variances, and mean loadings is the one with the highest values on the respective criterion. Random block selection serves as a benchmark. Any algorithm should perform better than random block selection in order to be worth using.

### Methods

In this simulation, an initial pool of blocks, yielding 240 pairwise comparisons, is reduced to one fourth (i.e., to 60 pairwise comparisons). The tests each measured five traits. Across the block pool, half of the pairwise item comparisons involved items that were keyed in different directions (i.e., one positive, one negative factor loading). I replicated the simulation study with all positively keyed items. Since the properties of ipsative trait estimates are quite different, the results were analyzed separately. Item intercepts $\mu _i$ were drawn from $U(-2,2)$. Item loadings $\lambda _i$ were drawn from *U*(.45, .95). Item uniquenesses $\psi _i^2$ were calculated as $1-\lambda _i^2$ (i.e., standardized item utilities were simulated). Errors were drawn from $N(0, \psi _i^2)$. The ranges of the item parameter distributions were larger than in Simulation Study 1 on *SE* accuracy so that the algorithms could improve trait recovery in comparison with random block selection. Information was calculated over a grid of points. Trait levels were set to $-1$, 0, and 1 and fully crossed for the five traits, thus yielding $3^5 = 243$ grid points. The only constraint was test length. A multivariate normal prior was used, with the covariances based on meta-analytic correlations between the Big Five (van der Linden et al. 2010, Table 1).

Three factors of the ATA problem were varied: First, the *target* information curve was either weighted, equal or a single point. For the weighted target, the target information was proportional to that of the block pool. For the equal target, all trait levels were weighted equally (i.e., the target surface was flat). The weighted and equal targets simulate the construction of a test for a general population (called population test). For the single target, the target was a single grid point, namely the vector of ones. The single target simulates the construction of a screening test with a cut-off point at the level of one for each trait (called screening test). Second, the *intercepts* were either ordered or random. For the ordered intercepts, the intercepts were first ordered by quartiles and then assigned to blocks so that within each block, the intercepts were from the same quartile of the intercepts distribution. The design was balanced so that each combination of traits had the same amount of blocks in each quartile. The ordered intercepts lead to a higher variance of information across blocks. For the random intercepts, the intercepts were randomly assigned to blocks. Third, *block size* was either two, three, or four. The number of pairwise comparisons was kept equal across block sizes. In the initial pool, there were 240 pairwise comparisons. Thus, for block sizes two, three, and four, the initial pool comprised 240, 80, and 40 blocks, respectively. The final tests each comprised one fourth of this, that is, 60, 20, and 10 blocks, respectively.

#### Algorithms

MIP based on trace For MIP based on the trace, a maximin criterion was chosen to select the combination of blocks so that the trace of the test information matrix was maximal, whereas, across grid points, it was close to proportional to the target trace. The MIP solver I used was lpSolve with the R package lpSolveAPI (lp_solve, Konis & Schwendiger 2020) as an interface (see Diao & van der Linden 2011, for an illustration of how to use lpSolveAPI for MIP with single-stimulus items).

Greedy algorithm based on variances For the greedy algorithm based on variances, for each block that was not in the current test, the sum of the sampling variances that was achieved by adding this block to the current test was calculated for each grid point. For the weighted target, the sum of the variances was weighted by the sum of the variances in the block pool for this grid point. The weighted or unweighted sum of the variances was then averaged across grid points, yielding the mean sum of the variances. The block with the lowest mean sum of the variances was added to the current test. This procedure was repeated until the final test length of one quarter of the block pool was reached.

Greedy algorithm based on determinant The greedy algorithm based on the determinant was identical to that based on the variances, except that the determinant of the test information matrix was used instead.

*Block* $$R^2$$

To obtain one value per block, block $R^2$ was averaged across traits. For the weighted target, instead of using a maximin criterion, block $R^2$ was weighted across grid points by the sum of the sampling variances in the block pool. The quarter of the blocks with the highest weighted mean block $R^2$ were selected.

Mean variances The posterior sampling variances were calculated for each block separately and averaged across traits. That is, the prior was added to block information (Eq. 8): ${\textbf{I}}_{jk}^\text {posterior} = {\textbf{I}}_{jk} + \Sigma ^{-1}$. For the weighted target, the mean variances were weighted across grid points by the sum of the mean variances in the block pool. The blocks with the highest weighted mean variances were selected.

Mean loadings For mean loadings, the blocks with the highest mean absolute loadings were selected.

Random block selection For random block selection, the blocks were selected randomly.

#### Procedure

Two hundred replications were conducted. All data simulation and analysis were implemented in R, using the same R packages as in Simulation Study 1 on *SE* accuracy, in addition to lpSolveAPI. First, item parameters were drawn. Second, information was estimated for the grid points. For the weighted and equal targets, the grid points were obtained by fully crossing the levels of $-1$, 0, and 1 for the five traits. For the single target, the grid point was the vector of ones. Third, a test was assembled involving each of the six algorithms. Fourth, trait and response vectors were drawn to later evaluate estimation accuracy. For the weighted and the single target, the trait vectors were drawn from a multivariate normal distribution with a mean vector of 0 and covariances that were based on meta-analytic correlations between the Big Five (Table 1) for 500 respondents. For the equal target, the grid points served as trait levels. There were 243 grid points. To achieve a sample size that was comparable to the weighted target, each grid point was duplicated, yielding 486 respondents. Responses for these respondents on the block pool were simulated. Fifth, the trait levels were estimated as MAP estimates for each of the four assembled tests on the basis of the true item parameters and the Big Five correlations.

Last, outcome measures were computed: To assess how well the test information target was approximated, the three optimization criteria were computed based on the assembled test. In addition, a measure of the difference between test information in the block pool versus the assembled test was computed. The details on these outcome measures are given in supplementary online material.

To assess trait recovery in the population test (weighted and equal targets), three outcome measures were calculated across the $n = 1, \dots , N$ respondents within each condition and replication: the correlation between the true and estimated traits $r(\theta , \hat{\theta })$, the RMSE (Equation 19, with $\xi =\theta$ and $m=n$), and the mean absolute bias (MAB):

26 $$\begin{aligned} MAB(\theta )&= \frac{\sum _{n=1}^{N}|\hat{\theta }_n - \theta _n|}{N}. \end{aligned}$$

To assess the screening test (single target), instead of trait recovery, sensitivity and specificity were calculated. Sensitivity was defined as the proportion of respondents correctly classified as having a trait level > 1. Likewise, specificity was defined as the proportion of respondents correctly classified as having a trait level < 1.

**Table 5:** Mean trait recovery by condition in simulation study 2 on test construction for the equal and weighted targets (population test).

Intercepts	Target	Algorithm	$r(\theta ,\hat{\theta })$		$r(\theta ,\hat{\theta })^2$		MAB		RMSE	
Random	Weighted	Greedy Variances	0.92	(0.01)	0.85	(0.03)	0.30	(0.03)	0.15	(0.03)
		Greedy Determinant	0.92	(0.02)	0.85	(0.03)	0.31	(0.03)	0.15	(0.03)
		MIP trace	0.92	(0.02)	0.85	(0.04)	0.31	(0.04)	0.16	(0.04)
		Block $R^2$	0.92	(0.02)	0.85	(0.03)	0.31	(0.03)	0.15	(0.03)
		Mean variances	0.90	(0.02)	0.82	(0.04)	0.33	(0.03)	0.19	(0.04)
		Mean loadings	0.91	(0.02)	0.84	(0.04)	0.32	(0.03)	0.17	(0.04)
		Random	0.88	(0.03)	0.78	(0.05)	0.37	(0.04)	0.22	(0.04)
	Equal	Greedy variances	0.89	(0.02)	0.80	(0.04)	0.29	(0.03)	0.13	(0.03)
		Greedy determinant	0.89	(0.02)	0.80	(0.04)	0.29	(0.03)	0.13	(0.03)
		MIP trace	0.89	(0.03)	0.79	(0.05)	0.30	(0.04)	0.14	(0.04)
		Block $R^2$	0.89	(0.03)	0.80	(0.04)	0.29	(0.03)	0.14	(0.03)
		Mean Variances	0.87	(0.03)	0.76	(0.05)	0.31	(0.03)	0.16	(0.03)
		Mean loadings	0.88	(0.03)	0.77	(0.05)	0.31	(0.04)	0.15	(0.03)
		Random	0.83	(0.04)	0.69	(0.06)	0.36	(0.04)	0.21	(0.04)
Ordered	Weighted	Greedy variances	0.93	(0.01)	0.87	(0.02)	0.29	(0.02)	0.14	(0.02)
		Greedy determinant	0.93	(0.01)	0.86	(0.02)	0.29	(0.02)	0.14	(0.02)
		MIP Trace	0.93	(0.01)	0.87	(0.03)	0.29	(0.03)	0.14	(0.03)
		Block $R^2$	0.93	(0.01)	0.86	(0.03)	0.29	(0.03)	0.14	(0.03)
		Mean variances	0.90	(0.04)	0.82	(0.06)	0.32	(0.04)	0.18	(0.06)
		Mean loadings	0.93	(0.02)	0.86	(0.03)	0.29	(0.03)	0.14	(0.03)
		Random	0.91	(0.02)	0.83	(0.04)	0.33	(0.03)	0.18	(0.04)
	Equal	Greedy variances	0.92	(0.01)	0.84	(0.03)	0.26	(0.02)	0.11	(0.02)
		Greedy determinant	0.91	(0.02)	0.84	(0.03)	0.26	(0.02)	0.11	(0.02)
		MIP trace	0.91	(0.02)	0.84	(0.04)	0.26	(0.03)	0.11	(0.02)
		Block $R^2$	0.91	(0.02)	0.83	(0.03)	0.26	(0.03)	0.11	(0.02)
		Mean variances	0.90	(0.03)	0.80	(0.05)	0.28	(0.03)	0.13	(0.03)
		Mean loadings	0.91	(0.02)	0.83	(0.03)	0.26	(0.03)	0.11	(0.02)
		Random	0.88	(0.03)	0.77	(0.05)	0.31	(0.04)	0.15	(0.04)

MAB = mean absolute bias, RMSE = root mean squared error, MIP = mixed integer programming.

Standard deviations are given in parentheses.

**Table 6:** Variance in trait recovery explained in % by algorithm, target and intercepts in simulation study 2 on test construction for the equal and weighted targets (population test).

Factor	$r(\theta , \hat{\theta })$	MAB	RMSE
Algorithm versus Random	13	18	15
Optimality versus Means	2	2	2
$R^2$ versus Mean Variances	3	3	4
Intercepts	10	12	8
Target	18	6	9
2 versus 3 and 4	1	5	1
3 versus 4	4	4	4
Algorithm versus Random $\times$ Intercepts	0	1	1
Target $\times$ Intercepts	1	1	1
Algorithm versus Random $\times$ 2 versus 3 and 4	0	0	1
Optimality versus Means $\times$ 2 versus 3 and 4	1	1	2
$R^2$ versus Mean Variances $\times$ 2 versus 3 and 4	2	2	2
2 versus 3 and 4 $\times$ Intercepts	1	1	2
2 versus 3 and 4 $\times$ Target	1	0	1
Residuals	40	42	46

MAB = mean absolute bias, RMSE = root mean squared error.

$r(\theta , \hat{\theta })$ was Fisher *Z* transformed.

All outcome measures were summarized via means and *SD*s by condition, and the explained variance for the contrasts between conditions was calculated in an ANOVA framework. For the ANOVA, $r(\theta , \hat{\theta })$ was Fisher-*Z*-transformed.

### Results

Three MIP models did not converge for a block size of two. In presenting the results, in line with RQ3, I focus on the differences between the algorithms and their interactions with the other design factors.

#### Trait Recovery of the Population Test

**Fig. 4:**
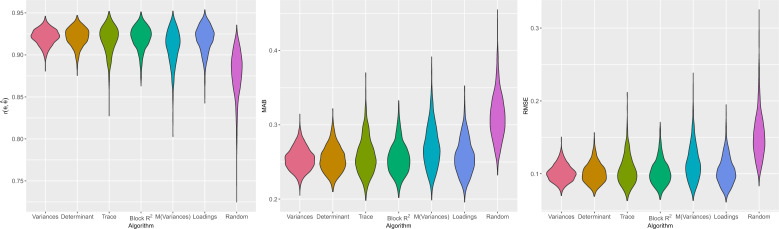
Trait recovery by algorithm, for a block size of three and the ordered intercepts and the equal target, in Simulation Study 2 on test construction. The bulge indicates the density, obtained by kernel density estimation. M = mean, MAB = Mean Absolute Bias, RMSE = Root Mean Squared Error.

Trait recovery was examined for the weighted and equal targets only (i.e., for the population test). Trait recovery was worse for random block selection (e.g., mean MAB = 0.34) than for the other algorithms together (mean MAB = 0.29, Table 5, Fig. 4), explaining 13% to 18% of the total variance (Table 6). Recovery was slightly worse for the algorithms based on means, that is, mean variances, mean block $R^2$, and mean loadings (e.g., mean MAB = 0.30) than for MIP based on the trace and the greedy algorithms based on the variances and on the determinant (mean MAB = 0.29). Moreover, recovery was worse for mean variances (e.g., mean MAB =.31) than for mean block $R^2$ (mean MAB =.29). However, these difference explained only 2% to 4% of the variance. These differences were higher for block size 2 than for block sizes 3 and 4, explaining between 1% and 2% of the variances, except for the difference between random block selection and the other algorithms. Descriptively, the variance in recovery was highest for mean variances, followed by MIP for the trace, and mean loadings (Fig. 4). Mean block $R^2$ performed most similar to the greedy algorithms based on the variances and on the determinant. The difference between random block selection and the other algorithms was slightly higher for the random intercepts.

#### Sensitivity and Specificity of the Screening Test

**Fig. 5:**
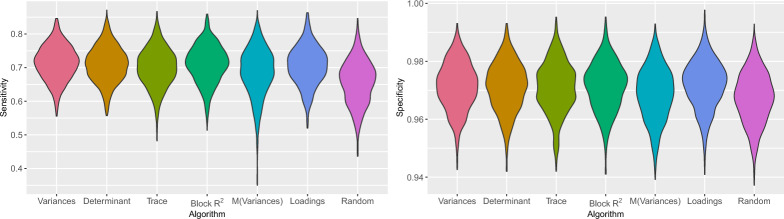
Sensitivity and specificity by algorithm, for a block size of three, the ordered intercepts, and the single target (screening test), in Simulation Study 2 on test construction. The bulge indicates the density, obtained by kernel density estimation. M = Mean.

Sensitivity and specificity were examined only for the single target (i.e., the screening test). Specificity was high and did not differ across the algorithms and conditions (mean =.97, SD =.01, Fig. 5, Table 7). Sensitivity was lower (mean =.68) and varied slightly across conditions. For the random intercepts, sensitivity was lower for random block selection (mean =.61), followed by mean variances and mean loadings (mean =.67), followed by the other algorithms (mean =.69). For the ordered intercepts, the differences between the algorithms were smaller. Here, sensitivity was lowest for random block selection (mean =.66), followed by the trace and mean variances (mean =.68) and the other algorithms (mean =.70).

**Table 7:** Variance in sensitivity and specificity explained in % by algorithm, intercepts and block size in simulation study 2 on test construction for the single target (screening test).

Factor	Sens.	Spec.
Algorithm versus random	7	1
Block $R^2$ versus mean variances	1	1
2 versus 3 and 4	3	2
3 versus 4	2	0
Intercepts	1	1
Algorithm versus random $\times$ Intercepts	1	0
2 versus 3 and 4 $\times$ Intercepts	1	0
Residuals	81	93

Sens = sensitivity, Spec = specificity.

#### Optimization Criteria

The detailed results for the optimization criteria are given in supplementary online material. In sum, block $R^2$ performed second best or best across all optimization criteria, together with the greedy algorithms based on the variances and on the determinant (Figure S4). In addition, descriptively, there were some interactions with the screening versus population test: MIP based on the trace performed worse than expected in the population test. The mean variances performed worse for the screening test than for the population test (Table S5, Figure S4).

#### Simulation Results with All Positively Keyed Items

The results of the simulation with all positively keyed items are shown in Tables S2–S7 and Figures S2–S5. In sum, with respect to the optimization criteria, the differences between the algorithms were similar (Tables S2 and S3). However, block $R^2$ performed clearly worse than the other algorithms (Table S3, Figure S2). The differences were more pronounced for block size two and for the random intercepts. With respect to recovery, for block size $B=2$, block $R^2$ performed much better than the other algorithms (Figure S4). In this condition, mean loadings and mean variances did not perform better than random block selection. For block sizes two and three, block $R^2$ showed higher variance than the other algorithms (Figure S3). With respect to sensitivity and specificity, the differences between the algorithms were even less pronounced than with mixed keyed items.

### Discussion

#### Performance of the Algorithms

The results of this simulation showed that the algorithms and information summaries performed better than random block selection and are thus worth using. However, with respect to most outcome measures, the differences between the performance of the algorithms were small.

The mean variances performed worst across several outcome measures. Therefore, they are not recommended. Probably, for a single block, the prior is too influential.

The mean loadings turned out to be a good alternative when the distribution of the item difficulties was around the population mean (i.e., for the population test). When the goal was to select items measuring a higher trait level than the mean of the item distribution (i.e., for the screening test), the mean loadings performed worse with respect to the optimization criteria. This is because they ignore the information in the intercepts. Thus, using the mean loadings as a proxy to block information can only be recommended when the items in the pool are (evenly) distributed around the target. The mean loadings have the advantage that they do not require any considerable computational effort (besides model fitting, which is needed for any method).

Block $R^2$ performed second best with respect to most outcome measures. Therefore, it is a good all-rounder that can be used for several purposes. Note that in this simulation, the balancing of items across traits was ideal. Using mean block $R^2$ across traits with unbalanced numbers of items per trait might result in primarily selecting blocks that include the less represented traits thus increasing the measurement precision for these traits. If this is not desired, it can be alleviated by constraining the numbers of items per trait or by weighing block $R^2$ across traits by the trait reliability.

The sum of the sampling variances and the determinant of the test information matrix performed quite well despite they were combined with a simple greedy algorithm. The performance of a greedy algorithm provides a lower bound estimate to that of a more elaborate heuristic. Thus, the variances and the determinant are promising information summaries for the development of a local search heuristic or a more elaborate constructive heuristic. With respect to most outcome measures, the determinant slightly outperformed the variances. In addition, computing the determinant is computationally less intensive than computing the variances because it does not involve matrix inversion.

The trace showed higher variance and performed worse in approximating the optimization criteria. This might be because it ignores the contribution from correlated traits. Although the maximin criterion implemented in the MIP algorithm should generally outperform the weighted criterion in the other algorithms (van der Linden 2005), this advantage was not visible in this simulation.

#### Sequential Versus Non-sequential Algorithms

In general, non-sequential algorithms, such as MIP algorithms, are preferred over sequential ones, such a greedy algorithms (van der Linden 2005). This is because they can find the optimal solution if it exists. Sequential algorithms can only find locally optimal solutions. There is no guarantee that consecutive locally optimal solutions lead to the final optimal solution. Interestingly, in this simulation, this advantage was not found. Rather, the sequential (greedy) algorithms outperformed the non-sequential (MIP) algorithm. Most likely, this was because the criteria used in the greedy algorithms (variances and determinant) outperformed the one used in MIP (trace). This is especially remarkable since the sequential algorithm used in this simulation was the simplest one, namely a greedy one. More sophisticated heuristics have been successfully used for other test construction problems (e.g., Kreitchmann et al. 2021; Olaru et al. 2015). In terms of formulating the test construction problem, MIP algorithms have the advantage to be most flexible, whereas local search heuristics are specifically tailored to a certain problem (van der Linden 2005).

#### Limitations

The composition of the block pool was rather ideal with all combinations of three out of the five traits occurring equally often and half of the pairwise comparisons between mixed keyed items. Varying the block pool or constraining the ATA problem should have similar effects on the performance of the algorithms. In previous simulations, both constrained and unconstrained ATA problems were simulated. This did not result in differences with respect to the performance of the algorithms. However, with even more variance in the block pool, which might be observed in empirical studies, constraints might be more effective.

This simulation examined only a limited set of conditions. Specifically, only five traits were simulated, and the correlations between the traits were not varied. Although these settings might be representative of some applied tests (e.g., Brown & Maydeu-Olivares 2011; Wetzel & Frick 2020), more research is needed on how well the methods examined perform under different test designs and for more complex ATA problems. Increasing the number of traits comes with computational challenges: Estimating block information for a single person with a block size of three and 15 traits took between 3.5 and 5.5 hr on the high-performance computing clusters I had access to. Thus, the run times are currently too high for a simulation. Nevertheless, block information for an empirical sample could be computed even for a large number of traits.

With all positively keyed items, the results were quite different for the optimization criteria and trait recovery. Therefore, more research is needed to investigate what drives the performance of the information summaries and ATA algorithms in these settings.

#### Recommendations for the Use of Block Information in Test Construction

When a test constructor does not want to compute information at all, the mean absolute loadings are a good proxy but only as long as the item intercepts are evenly distributed around the target’s mean. When a test constructor wants to visually examine the blocks of a fixed test, block $R^2$ is recommended since it performed well with respect to most outcome measures. When the test construction problem includes constraints, only the trace with an MIP algorithm and block $R^2$ can be used so far since the simple greedy heuristic presented here does not allow to include constraints. When a greedy heuristic is sufficient, the determinant performed slightly better than the variances. For test designs with all positively keyed items, no clear recommendation can be given at the moment since the performance of the information summaries differed qualitatively between the optimization criteria and trait recovery. In addition, if certain traits are more relevant to the assessment, it can be worth applying several algorithms and choosing the solution that best maximizes reliability and validity for the more relevant traits (see Empirical Application).

## Empirical Application

To illustrate the use of block information with empirical data, the algorithms for automated test assembly were applied to develop short versions of the Big Five Inventory 2 (BFI-2; Soto & John 2017) in the MFC format. In addition, this application illustrates how to use constraints in addition to test length, for example, on the number of items per trait, in an ATA problem for MFC test construction. In order to include constraints, I omitted the greedy algorithm. In this case, including a prior on the trait covariances was not necessary and was therefore also omitted.

### Methods

#### Sample and Procedure

This is a secondary analysis of a data set that was collected for another study (Kupffer et al. 2022). Data were collected in an online survey via Prolific Academic https://www.prolific.co. One hundred and twenty-two participants were excluded because they failed an instructed response block and 16 participants were excluded because they responded 2 *SD* faster than the mean. The final sample consisted of 1,031 participants. The mean age was 36 years (*SD* = 12). Sixty-seven percent were female, and 0.5% were transgender. The participants responded to six MFC questionnaires with a block size of three, out of which the ones in the present analyses were presented first, second, and fourth.

#### Measures

The BFI-2 was originally a rating scale questionnaire for measuring the Big Five personality traits: neuroticism, extraversion, openness, agreeableness, and conscientiousness. Each trait is assessed with 12 items. For the purpose of the study for which the data were collected, an MFC version with a block size of three was constructed. This version is henceforth referred to as the full version. In the full version, all 10 combinations of three out of five traits occur twice. When neuroticism is recoded to emotionality, six (out of 20) blocks are mixed keyed (i.e., they contain one negatively keyed and two positively keyed items or two negatively keyed and one positively keyed item). The BFI-2 items and the composition of the MFC version are shown in Table S5 in supplementary online material.

The Big Five Triplets (Wetzel & Frick 2020) make up an MFC questionnaire measuring the Big Five personality traits with a block size of three. The number of items per trait ranges from seven to sixteen. The HEXACO-60 (Ashton & Lee 2009) is originally a rating scale questionnaire measuring the HEXACO personality traits: honesty–humility, emotionality, extraversion, agreeableness, conscientiousness, and openness. Each trait is assessed with 10 items. The HEXACO-60 was administered in an MFC version with a block size of three.

#### Automated Test Assembly

Short versions of 10 blocks were constructed with constraints such that the numbers of items per trait were equal, at least half of the pairwise comparisons across the test were between differently keyed items (i.e., at least eight mixed keyed blocks), and there was at least one negatively keyed item per trait. The target information was proportional to the information in the full version. Three short versions were assembled using MIP with the trace, mean block $R^2$, and mean loadings. For the mean loadings, the estimated loadings were first standardized by dividing them by the variance of the item utilities: $\lambda _i^{std} = \lambda _i/(\lambda _i^2*{Var}(\varvec{\Theta }) + \psi _i^2)$, where ${Var}(\varvec{\Theta })=1$.

#### Analysis

The Thurstonian IRT model was fit to the full test in Mplus (Muthén & Muthén 1998). The model fit was good according to the RMSEA (.033) and close to acceptable according to the SRMR (.085). Based on the estimated parameters, the block information was computed for a grid of points, obtained by fully crossing the trait levels $-1$, 0, and 1 for the five traits. The item parameter estimates and block information summaries are shown in Table S5. Then, the short versions were assembled. For each short version, the MAP estimates for the empirical sample were obtained with the estimated trait correlations from the full test as a prior. Empirical reliabilities were calculated for all versions, using the following formula with observed *SE*s:

27 $$\begin{aligned} Rel_{emp} = \frac{Var(\varvec{\theta })}{Var(\varvec{\theta }) + Mean(\mathbf {SE_\theta ^2)}}. \end{aligned}$$

Then, the square roots of the empirical reliabilities were Fisher-Z-transformed, and the differences in the reliabilities were compared between the full version and the three short versions. In addition, MAP estimates were obtained for the Big Five Triplets and the HEXACO. Correlations were calculated between the MAP estimates for the BFI-2 versions on the one hand and the Big Five Triplets and the HEXACO on the other hand. The differences between the Fisher-Z-transformed correlations were compared between the full version and the three short versions. I focused on the correlations between the same traits assessed with the BFI-2 and the Big Five Triplets, between similar traits assessed with the BFI-2 and the HEXACO, and between BFI-2 agreeableness and HEXACO honesty–humility. For these, medium to large correlations were expected on the basis of the literature (Thielmann et al. 2022).

### Results

#### Block Compositions

All three MIP models converged. Table S5 shows which blocks were selected in which version. Three blocks were selected in all three short versions. The versions based on block $R^2$ and the mean loadings had one and two blocks that were unique to this version, respectively. The versions based on the trace and on block $R^2$ contained eight out of 10 combinations of traits. Thus, in these version, two trait combinations occurred twice. In the version based on mean loadings, all 10 trait combinations occurred. All three short versions had eight mixed keyed blocks. That is, the lower limit of the mixed keyed blocks was selected in all three short versions. Usually, three or four (out of six) items per trait were negatively keyed. The short version based on block $R^2$ had only two negatively keyed extraversion and openness items and the version based on mean loadings had only one negatively keyed extraversion item.

#### Empirical Reliabilities

The empirical reliabilities for all versions and the correlations with the full version are shown in Table 8. The decreases in reliability compared with the full version were mostly small effects according to Cohen (1992,.10 $< |$difference in Fisher *Z*$|<$.30). With MIP based on the trace, the decrease in the empirical reliability of openness was on the border of a medium effect (0.30). With mean loadings, the decreases in the empirical reliabilities of openness and agreeableness were medium effects (0.37 and 0.32, respectively). Overall, the decreases were slightly larger with mean loadings than with MIP based on the trace and block $R^2$.

**Table 8:** Empirical reliabilities and correlations with the full version for MAP estimates from the reduced versions of the Big Five Inventory 2.

Algorithm	Empirical reliability					Correlation with Full				
	N	E	O	A	C	N	E	O	A	C
MIP trace	0.69	0.67	0.49	0.58	0.63	0.93	0.96	0.9	0.9	0.93
Block $R^2$	0.7	0.64	0.53	0.59	0.61	0.93	0.94	0.9	0.92	0.93
Mean loadings	0.7	0.61	0.44	0.53	0.62	0.93	0.92	0.79	0.88	0.92
Full	0.81	0.73	0.68	0.72	0.74					

N = neuroticism, E = extraversion, O = openness, A = agreeableness, C = conscientiousness, MIP = mixed integer programming, Full = full version.

**Table 9:** Convergent validities of MAP estimates for the versions of the Big Five Inventory 2.

Algorithm	Big Five Triplets					HEXACO					
	N	E	O	A	C	Emo	Ext	Ope	Agr	Con	A-HH
MIP trace	0.62	0.63	0.45	0.25	0.24	0.49	0.5	0.58	0.42	0.54	0.48
Block $R^2$	0.64	0.62	0.45	0.29	0.19	0.5	0.5	0.59	0.44	0.52	0.48
Mean loadings	0.61	0.58	0.46	0.25	0.21	0.48	0.51	0.43	0.5	0.53	0.49
Full	0.69	0.65	0.5	0.35	0.34	0.59	0.53	0.62	0.53	0.56	0.49

N = neuroticism, E = extraversion, O = openness, A = agreeableness, C = conscientiousness, Emo = emotionality, Ext = extraversion, Ope = openness, Agr = agreeableness, Con = conscientiousness, HH = honesty–humility, MIP = mixed integer programming, Full = full version.

#### Construct Validities

The correlations between the same or similar traits assessed with the BFI-2 versions in comparison with the Big Five Triplets and the HEXACO are shown in Table 9. For the same traits assessed with the BFI-2 and the Big Five Triplets, there were small decreases in the correlations for neuroticism, agreeableness, and conscientiousness when MIP based on the trace was applied. With mean block $R^2$, there was a small decrease in the correlation for conscientiousness. With mean loadings, there were small decreases in all the correlations besides the one for openness. For similar traits assessed with the BFI-2 and the HEXACO, there were small decreases in the correlations for neuroticism and agreeableness when MIP based on the trace and mean block $R^2$ were used. With mean loadings, there were small decreases in the correlations for neuroticism and openness.

### Discussion

To illustrate the application of block information, three short versions of the Big Five Inventory 2 were constructed. All three versions were fairly balanced regarding trait combinations and item keying, although the versions based on the trace and on block $R^2$ were less balanced than the full questionnaire, as to be expected. The reliabilities decreased slightly, which is to be expected with half the number of items. The decreases were largest for mean loadings. For the validities, however, most of the decreases were marginal. Again, the decreases in the validities did not vary systematically between the algorithms. Tentatively, for the Big Five Triplets, the decreases in the validities were largest for mean loadings. The differences in the validities between the algorithms can probably be attributed to the differences in the reliabilities of the trait estimates. Recall that the validities were based on observed correlations of the trait estimates and were therefore not corrected for measurement error. Overall, mean loadings performed slightly worse but the trace and block $R^2$ performed on a par with each other. Thus, the decision of which short version to choose should be based on which traits are most relevant to the assessment. For example, in the current application, when the focus is on agreeableness, the short version based on block $R^2$ would be preferred because it showed the highest reliabilities and validities for this trait. Likewise, when the assessment focus is on conscientiousness, the version based on the trace should be preferred. Thus, when the computational effort allows it, several short versions could be constructed and the one that best assesses the most relevant traits could be chosen. Alternatively, the target could be adapted to weigh the traits by their relevance.

## General Discussion

In this manuscript, I investigated the accuracy of Fisher information in Thurstonian IRT models and how it can be used for test construction. In the first part, I focused on the accuracy on the test level. A simulation study showed that the observed and expected standard errors based on the block information were similarly accurate. The independence likelihood underestimated the standard errors when local dependencies were present with block sizes $>2$.

In the second part, I focused on the accuracy on the block level by simulating test construction based on block information. Because Fisher information for a block is multidimensional, I proposed to use several indices to summarize block information into a scalar: block $R^2$, the determinant and the trace of the information matrix, and the sum of the sampling variances. In a simulation study, the information summaries in conjunction with different test assembly algorithms showed small differences depending on the outcome considered, but they performed overall on a par with each other. Finally, an empirical application illustrated how the block information summaries can be used to automatically construct a short version of the Big Five Inventory 2 in the MFC format. In the following, I outline possible applications of block information in research and practice.

### Statistical Improvements

With Fisher information on the block level, unbiased expected and observed *SE*s can be obtained for block sizes $>2$ (Yousfi 2020). Although the overestimation of reliability based on information for binary outcomes of pairwise comparisons is small (Brown & Maydeu-Olivares 2011; Frick et al. 2023), it increases as block size increases. Block information allows users to calculate unbiased information summaries that can be used in test construction.

Future research could investigate other definitions of information, such as Kullback–Leibler information. Kullback–Leibler information is a scalar regardless of the number of traits assessed in the test. Therefore, it can be used to avoid the complications coming from a non-invertible matrix. It has been used successfully both in computerized adaptive testing (e.g., Mulder & van der Linden 2009) and in automated test assembly (e.g., Debeer et al. 2020). However, obtaining Kullback–Leibler information is computationally more demanding because it involves at least one more step of integration and still needs to be made estimable for Thurstonian IRT models (Lin 2020).

Admittedly, computing block information is computationally intensive. Current computing capacities prevented me from including simulations with a large number of traits, although MFC tests with, for example, 15 traits are quite prevalent (e.g., Drasgow et al. 2012; Holdsworth 2006; Peterson et al. 1999). Computing block information with a large number of traits is still possible for empirical samples. However, the accuracy of the estimation and of test assembly algorithms in this setting currently cannot be verified in simulation studies.

### Focus on the Block Level

In this manuscript, I propose to estimate Fisher information in the Thurstonian IRT model on the block level and examine its performance. This is in contrast to previous approaches on estimating information about the latent traits that focus on pairwise item comparisons. Similarly, the test assembly algorithms investigated here focus on selecting blocks as fixed units in contrast to those algorithms that re-assemble possible item comparisons (Kreitchmann et al. 2021, 2023; Lin 2020). There are several reasons for focusing on the block level and treating blocks as fixed units: First, a focus on the block level in comparison with the item level better reflects the response options available to participants and thus captures the relative nature of MFC responses.

Second, relatedly, MFC tests have an inseparable design. Thus, all traits measured in a block mutually interact to influence ranking preferences and, correspondingly, Fisher information. As illustrated in the section on block $R^2$ plots, calculating information summaries on the block level can account for and visualize those mutual influences.

Third, if items within a block interact, blocks should be treated as fixed in test construction. The estimation of Thurstonian IRT models became possible when rank orders were recoded as binary outcomes whose dependencies could be modeled in a structural equation framework (Brown & Maydeu-Olivares 2011; Maydeu-Olivares 1999; Maydeu-Olivares & Brown 2010). This might tempt test constructors to treat item pairs as the unit of analysis. However, items in MFC blocks have sometimes been observed to function differently between different block compositions (Lin & Brown 2017) or response contexts, for example, simulated low- and high-stakes contexts (Lee & Joo 2021). Block information accounts for all item parameters in a block simultaneously. At least as long as the extent of item interactions and item parameter invariance between different compositions of items to blocks is unclear, a focus on the block level appears to be a useful supplement.

### Investigating the MFC Format

Block-level Fisher information can yield further insights into how item content and statistical peculiarities of the MFC format influence the precision of trait estimates.

An example for this is item keying. In simulations with MFC tests comprised of all positively keyed items, trait recovery was decreased (Bürkner et al. 2019; Schulte et al. 2021) and the trait estimates showed ipsative properties in almost all cases (Brown & Maydeu-Olivares 2011; Bürkner et al. 2019; Frick et al. 2023). Ipsative trait estimates cannot be compared between persons, and they bias correlation-based analyses such as factor structures or validity coefficients (Brown & Maydeu-Olivares 2013; Clemans 1966; Hicks 1970). In practice, MFC tests with all positively keyed items are still used, although they cannot be recommended on the basis of their statistical properties. One reason for their use might be that researchers have argued that blocks with mixed keyed items are more fakable because the items that are positively keyed toward desirable traits stick out (Bürkner et al. 2019). To date, there is little research comparing the fakability of mixed versus equally keyed blocks. One study showed that an MFC test in which the item blocks were matched for social desirability was less fakable than a rating scale version of the same test although the blocks were mixed keyed (Wetzel et al. 2021). In addition, recent research showed that matched undesirable blocks were more prone to faking than matched desirable blocks (Fuechtenhans & Brown 2022). Thus, a closer look into item desirability beyond item keying might be worthwhile. Comparing block information between mixed and equally keyed blocks might yield further insights into how item keying contributes to the recovery of normative trait levels.

Moreover, differences in item social desirability might lead to certain rank orders being more frequent. For example, it has been reported that agreement about which rank order should be preferred increased the more the items within the blocks differed in their social desirability (Hughes et al. 2021). If certain rank orders are more frequent due to socially desirable responding, the whole block might be less informative with respect to the content traits. Future empirical studies could investigate the effect of item matching on the magnitude of block information.

### Benefits for MFC Test Assembly

The information summaries investigated here can be used to assemble MFC tests that maximize the precision of trait estimation. For manual test assembly, block information is easier to interpret and incorporate than standardized item loadings, which may differ by binary outcomes (e.g., Wetzel & Frick 2020).

Further, the current simulations showed that block information can be used for the automated assembly of fixed tests and illustrated how to do so. Considering the complexity of assembling MFC tests, including the balancing of traits, item keying, and item desirability, automated test assembly might prove particularly valuable. Examining minimal restrictions for test composition, the current simulations serve as a proof of concept that shows that the block information summaries can be used for ATA. The full advantages might be observed with more complex restrictions and test information goals, and more sophisticated heuristics.

Lastly, the block information summaries can be used in computerized adaptive testing, where tests are assembled for each participant, on the basis of their answers. In later stages of computerized adaptive testing, the sum of the sampling variances and the determinant of the test information matrix can be used and might be preferable. These information summaries performed best in a simulation on computerized adaptive testing where MFC blocks were assembled from separate items (Lin 2020).

A drawback of using information for blocks instead of items is that whole blocks have to be removed from the item pool. The selection of whole blocks requires more items and therewith more time for participants and more research funds than newly assembling blocks from separate items. Future research and applications will show how practicable and necessary this procedure is.

In this manuscript, I propose to estimate Fisher information for multidimensional forced-choice blocks that are modeled with the Thurstonian IRT model on the block level. I investigated the effect of neglecting local dependencies on standard errors and presented and evaluated several ways to summarize the information matrix for test construction. I hope this manuscript will improve the construction of MFC tests and encourage further investigation of their properties.

### Supplementary Information

Below is the link to the electronic supplementary material.

Supplementary file 1 (pdf 1111 KB)

## Footnotes

1 For blocks of three items, the instruction to identify which item is preferred the most and the least yields the same data format.

2 For an example of how to estimate the thresholds with this restriction, see the Mplus (Muthén & Muthén 1998) script in MFCblockInfo (Frick 2022).

3 It is also possible to include equality constraints (i.e., $\sum _{k=1}^{K}c_{kn} x_k = d_n$). However, for quantitative attributes, they may often lead to infeasible solutions (van der Linden 2005) which is why I chose the formulation with inequality constraints here.
