# Supplementary material for: Estimating and Using Block Information in the Thurstonian IRT Model
Source: Psychometrika. 2023 Aug 28;88(4):1556–89. doi: 10.1007/s11336-023-09931-8 (PMC10656335; doi:10.1007/s11336-023-09931-8)
Supplement: Supplementary file 1 — (pdf 1111 KB) [file 11336_2023_9931_MOESM1_ESM.pdf]

Online Supplementary Material for

Estimating and Using Block Information in the Thurstonian IRT Model

Online Supplementary Material for

Estimating and Using Block Information in the Thurstonian IRT Model

Simulation on Standard Error Accuracy - Additional Results

These are the results for the mean bias (MB) and root mean square error (RMSE) of the latent trait estimates and for their empirical standard errors (*SE*s).

Overall, the bias in the trait estimates was lower and the *SE*s were smaller for medium trait levels and for the long test (Figure S1). For example, for the short test, the RMSE ranged from 0.22 to 1.14; for the long test, it ranged from 0.17 to 0.79. The ML estimator showed a slight outward bias (e.g., mean MB($\theta = 2$) = 0.13), whereas the MAP estimator showed a more pronounced inward bias (e.g., mean MB($\theta = 2$) = -0.40, Figure S1). Both the MB and RMSE were smaller for high loadings and for the MAP estimator. The RMSE was especially high for low loadings combined with the ML estimator. Further, the bias increased slightly with block size (with mean RMSEs of 0.40, 0.44, and 0.48 for block sizes 2, 3, and 4, respectively). This is because the number of pairwise comparisons was kept constant across block sizes, which implies that local dependencies increase and recovery decreases slightly. Moreover, for block sizes 3 and 4, for the MAP estimator, there was a small difference in the amount of bias between the genuine and the independence likelihood. This is because the prior is given too little weight because the independence likelihood is higher than the genuine one.

For the ML estimator, the empirical *SE*s were smaller for medium trait levels (Figure S1). For the MAP estimator, the empirical *SE*s were similar across trait levels, which might be attributable to the inward bias of the estimates. The empirical *SE*s were highest for the ML estimator when combined with low loadings, similar to the RMSE. For both estimators, the empirical *SE*s were smaller for trait levels of ± 2 than for ± 1.5. This might be due to the box constraints: For true trait levels of ± 2, many estimates were ± 3, resulting in smaller standard deviations of the estimates (i.e., the empirical *SE*s). Specifically, for the MAP estimator, for true trait levels of ± 2, the box constraint was only activated in 1.2% of the simulated tests, for at most 2/500 responses. For the ML estimator, for true trait levels of ± 2, it was activated in all the simulated tests for a mean of 53/500 ($SD = 41$) responses. Similar to the RMSE, the empirical *SE*s increased with block size, and there was a small difference in the empirical *SE*s between the genuine and the independence likelihood for the MAP estimator and block sizes 3 and 4.

In the main text, results were reported for the *SE*s computed at the true trait value. In general, similar results were observed when the *SE*s were computed at the trait estimate (Tables S1 and S2, Figures S2 and S3). The results differed for the RMSE of the *SE*s at extreme trait values ($\theta = \pm 2$). Here, the RMSE of the *SE*s was smaller at the true trait value as compared to the estimate. This was probably because the ML estimator pulls the values away from the mean.

Table S1

Means of bias for information-based standard errors computed at the trait estimate by condition in simulation study 1 on standard error accuracy

Method	Blocksize	Likelihood	Estimator	MB		RMSE	
expected	2	genuine	ML	-0.01	(0.03)	0.05	(0.04)
			MAP	0.04	(0.03)	0.05	(0.03)
		independence	ML	-0.01	(0.03)	0.05	(0.04)
			MAP	0.04	(0.03)	0.05	(0.03)
	3	genuine	ML	-0.01	(0.04)	0.06	(0.05)
			MAP	0.05	(0.04)	0.05	(0.04)
		independence	ML	-0.05	(0.04)	0.08	(0.05)
			MAP	0.01	(0.04)	0.03	(0.02)
	4	genuine	ML	-0.01	(0.06)	0.07	(0.07)
			MAP	0.06	(0.05)	0.06	(0.04)
		independence	ML	-0.09	(0.06)	0.12	(0.06)
			MAP	-0.02	(0.04)	0.05	(0.02)
observed	2	genuine	ML	0.00	(0.03)	0.05	(0.05)
			MAP	0.05	(0.04)	0.05	(0.04)
		independence	ML	0.00	(0.03)	0.05	(0.05)
			MAP	0.05	(0.04)	0.05	(0.04)
	3	genuine	ML	0.00	(0.05)	0.06	(0.06)
			MAP	0.06	(0.04)	0.06	(0.04)
		independence	ML	-0.05	(0.05)	0.08	(0.05)
			MAP	0.01	(0.04)	0.04	(0.03)
	4	genuine	ML	0.00	(0.06)	0.07	(0.07)
			MAP	0.06	(0.05)	0.07	(0.05)
		independence	ML	-0.09	(0.06)	0.12	(0.06)
			MAP	-0.02	(0.04)	0.05	(0.02)

Note. MB = Mean Bias, RMSE = Root Mean Squared Error, ML = Maximum Likelihood, MAP = Maximum a posteriori. Standard deviations are given in parentheses.

Table S2

Variance in bias for information-based standard errors computed at the trait estimate explained in % by the manipulated factors in simulation study 1 on standard error accuracy

Factor	MB	RMSE
estimator	23	5
length	2	15
likelihood	12	0
blocksize	5	5
loadings×estimator	2	1
estimator×length	2	1
loadings×likelihood	1	0
estimator×likelihood	0	3
estimator×blocksize	0	3
likelihood×blocksize	8	0
loadings×estimator×likelihood	0	1
loadings×likelihood×blocksize	1	0
estimator×likelihood×blocksize	0	2
loadings×estimator×likelihood×blocksize	0	1
Residuals	43	61

Note. MB = Mean Bias, RMSE = Root Mean Squared Error. Expected vs. observed explained less than 1% of variance.

Discussion

As to be expected, the results of this simulation showed that the bias was lower and the *SE*s were smaller for medium trait levels, longer tests, and higher loadings. Thus, higher loadings and longer tests are recommended because both the trait estimates and their *SE*s are more accurate. Both the bias and the *SE*s of the trait estimates increased with block size. This is because the amount of information was kept constant across block sizes on the level of the binary outcomes of pairwise comparisons. However, the comparisons do not contribute fully independent information (Brown & Maydeu-Olivares, 2011; Yousfi, 2018). Therefore, in this design, recovery decreases with block size. However, this effect was so small that it is probably negligible in practice. Similarly, the difference between the genuine and the independence likelihood was more pronounced as the block size increased.

Regarding the comparison of estimators, especially with small loadings, the *SE*s were more accurate for the MAP estimator than for the ML estimator. Similarly, on the basis of a comparison of several trait estimators under various test designs with a large multivariate normal sample, Lin (2020) recommended that the MAP estimator be used. In the current

simulation, the accuracy of the *SE*s for the MAP estimator with low loadings and short tests was underestimated by the information methods; that is, the estimated *SE*s were larger than the empirical *SE*s. Thus, the advantage that the MAP estimator offers with respect to precision might not be detectable in empirical applications.

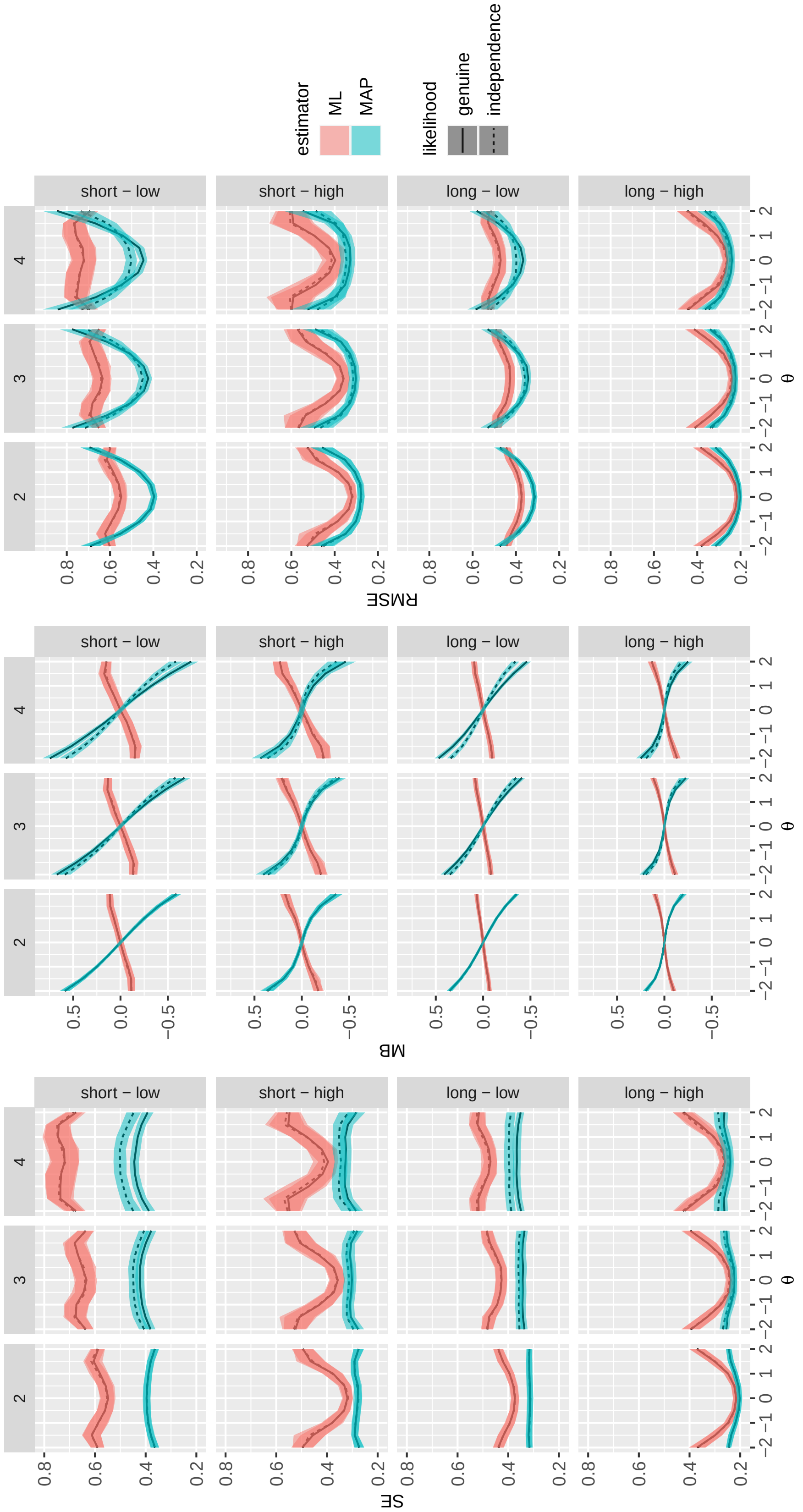

Figure S1. Trait recovery and empirical *SE*s in Simulation Study 1 on standard error accuracy. Shaded areas show ±1 *SD* around the mean (line). SE = empirical Standard Error, MB = Mean Bias, RMSE = Root Mean Square Error, ML = Maximum Likelihood, MAP = Maximum a Posteriori.

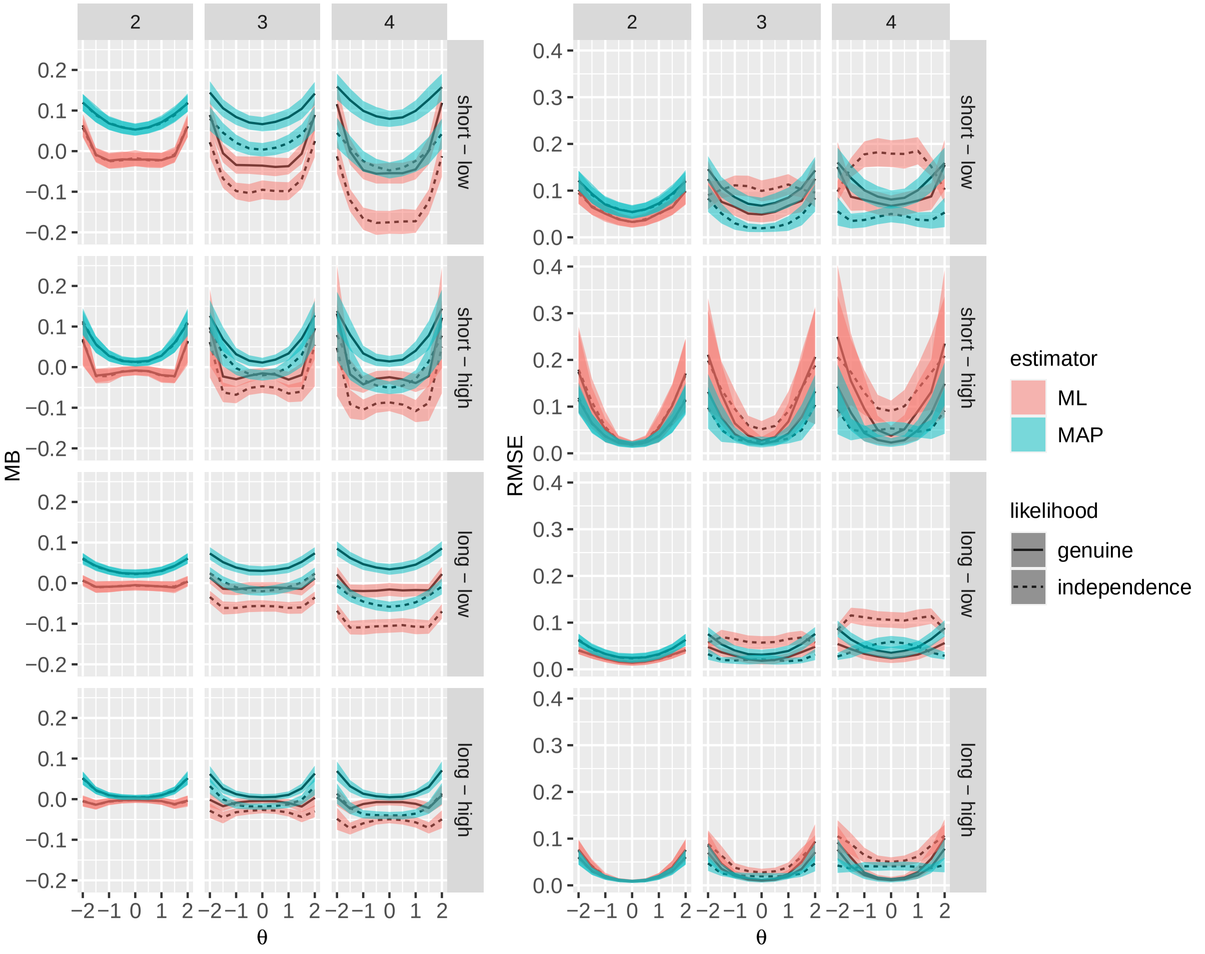

Figure S2. Bias for the observed standard errors computed at the trait estimate in Simulation Study 1 on standard error accuracy. Shaded areas show ±1 *SD* around the mean (line). MB = Mean Bias, RMSE = Root Mean Square Error, ML = Maximum Likelihood, MAP = Maximum a Posteriori.

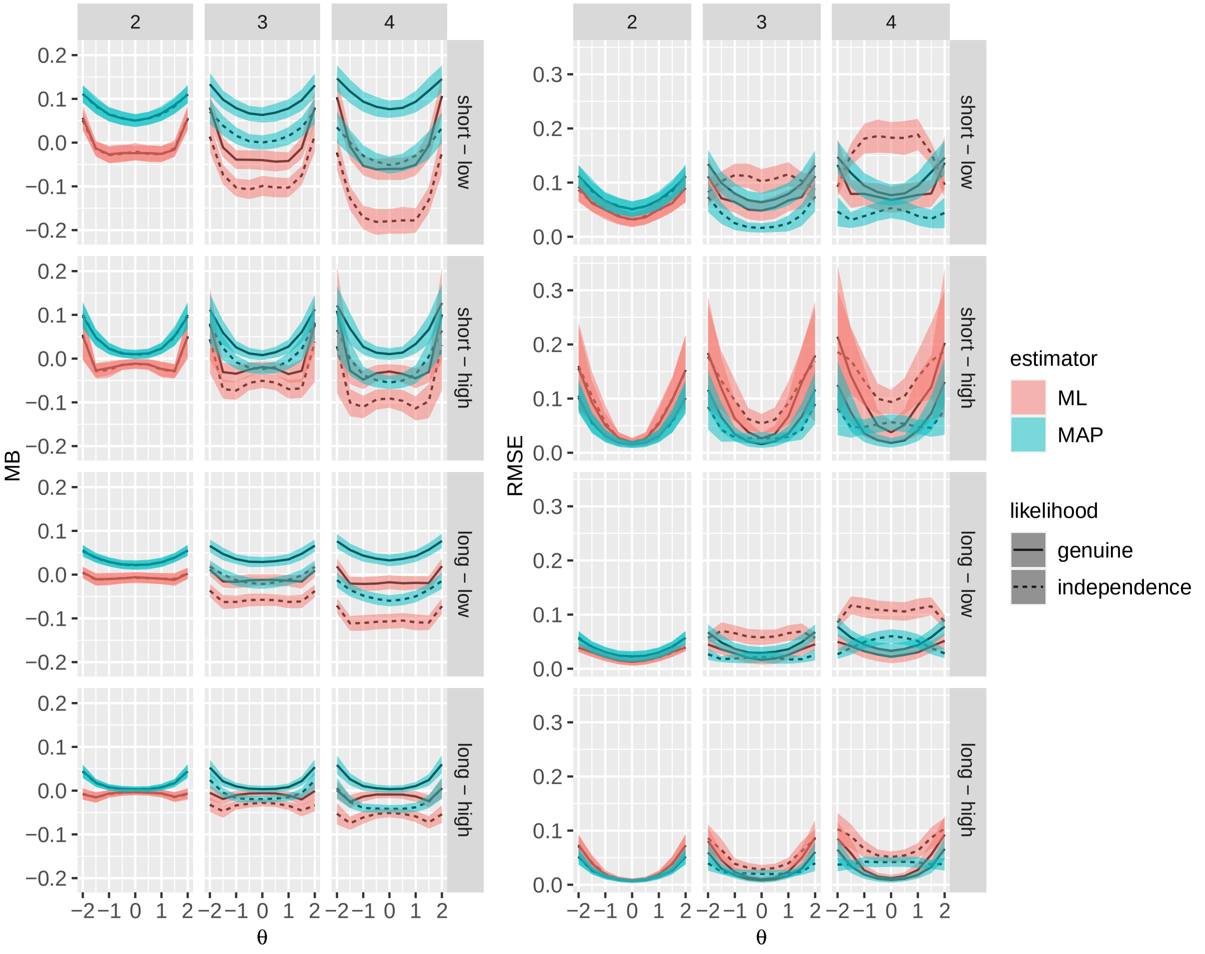

Figure S3. Bias for the expected standard errors computed at the trait estimate in Simulation Study 1 on standard error accuracy. The top row shows results for the short test (20 blocks) and the bottom row shows results for the long test (40 blocks). Shaded areas show $\pm 1SD$ around the mean (line). MB = Mean Bias, RMSE = Root Mean Square Error, ML = Maximum Likelihood, MAP = Maximum a Posteriori.

Simulation on Test Construction - Additional Results

Table S3

Mean sensitivity and specificity by condition in simulation study 2 on test construction for the single target (screening test)

Intercepts	Algorithm	Sensitivity		Specificity	
Random	Greedy Variances	0.70	(0.07)	0.97	(0.01)
	Greedy Determinant	0.69	(0.07)	0.97	(0.01)
	MIP Trace	0.69	(0.08)	0.97	(0.01)
	Block R^2	0.69	(0.07)	0.97	(0.01)
	Mean Variances	0.67	(0.07)	0.97	(0.01)
	Mean Loadings	0.68	(0.07)	0.97	(0.01)
	Random	0.61	(0.08)	0.97	(0.01)
Ordered	Greedy Variances	0.71	(0.06)	0.97	(0.01)
	Greedy Determinant	0.70	(0.06)	0.97	(0.01)
	MIP Trace	0.68	(0.06)	0.97	(0.01)
	Block R^2	0.71	(0.06)	0.97	(0.01)
	Mean Variances	0.68	(0.07)	0.97	(0.01)
	Mean Loadings	0.70	(0.06)	0.97	(0.01)
	Random	0.66	(0.07)	0.97	(0.01)

Note. MIP = Mixed Integer Programming. Standard deviations are given in parentheses.

Optimization Criteria

The criteria were less well optimized for random block selection (e.g., mean sampling variances = 0.89, Table S5, Figure S5) than for the other algorithms together (mean sampling variances = 0.59), explaining between 7% (Determinant) and 48% (Frobenius Norm) of the variance (Table S4). Except for the trace, the difference between random block selection and the other algorithms was higher for the random intercepts (e.g., mean difference in variances = 0.36 versus 0.22), explaining between 1% and 2% of the variance. Except for the variances, it was also higher for block size 2 than block sizes 3 and 4 (e.g., mean differences in Frobenius norm = 0.41 versus 0.29), explaining between 1% to 4% of the variance.

The criteria were better optimized with the information-based algorithms (e.g., mean sampling variances = 0.58) than with the mean loadings (mean sampling variances = 0.63), explaining between 1% to 3% of the variance. For the determinant and the Frobenius norm, this difference was higher for the screening test (single target). Descriptively, the variances and the determinant performed best at optimizing their own criteria, followed by block R^2

(Figure S5, Table S5). The trace was best optimized by the MIP algorithm based on the trace in the screening test (single target), followed by block R^2. In the population test (weighted and equal targets), it was best optimized by block R^2, followed by the determinant. The Frobenius norm was smalles for the greedy algorithms based on the variances and on the determinant and for block R^2.

Table S4

Variance in optimization criteria explained in % by algorithm, target, intercepts and block size in simulation study 2 on test construction

Factor	Var.	Det.	Trace	Frob.
Algorithm vs. Random	32	7	18	48
Info vs. Mean Loadings	1	1	0	2
Optimality vs. Means	1	1	1	3
R^2 vs. Mean Variances	2	4	2	6
Population vs. Screening	2	2	7	5
Weighted vs. Equal	5	4	0	0
2 vs. 3 and 4	17	30	24	6
3 vs. 4	4	1	3	0
Intercepts	17	7	27	9
Algorithm vs. Random × 2 vs. 3 and 4	0	4	2	1
Optimality vs. Means × 2 vs. 3 and 4	0	2	0	1
R^2 vs. Mean Variances × 2 vs. 3 and 4	0	4	1	2
Population vs. Screening × 2 vs. 3 and 4	0	2	0	1
Weighted vs. Equal × 2 vs. 3 and 4	0	2	0	0
Algorithm vs. Random × Intercepts	2	1	0	1
Population vs. Screening × Intercepts	3	0	4	1
Weighted vs. Equal × Intercepts	1	3	0	0
2 vs. 3 and 4 × Intercepts	0	3	1	2
Residuals	10	12	7	6

Note. Var = Variances, Det = Determinant, Frob = Frobenius.

Table S5

Mean optimization criteria by condition in simulation study 2 on test construction

Intercepts	Target	Algorithm	Variances		Determinant		Trace		Frobenius Norm	
Random	Population	Greedy Variances	0.63	(0.12)	47418.14	(41522.78)	48.73	(11.21)	1.18	(0.10)
		Greedy Determinant	0.63	(0.12)	49430.36	(43313.42)	49.24	(11.35)	1.18	(0.09)
		MIP Trace	0.66	(0.13)	38784.96	(33473.27)	47.56	(10.59)	1.21	(0.09)
		Block R^2	0.63	(0.12)	48283.07	(42393.91)	49.39	(11.35)	1.18	(0.10)
		Mean Variances	0.74	(0.08)	17797.98	(9631.66)	42.17	(6.47)	1.32	(0.04)
		Mean Loadings	0.71	(0.11)	24848.31	(17826.53)	42.21	(7.21)	1.28	(0.05)
		Random	1.00	(0.12)	3681.92	(2147.08)	28.58	(3.84)	1.56	(0.05)
	Screening	Greedy Variances	0.58	(0.12)	101841.56	(112026.51)	46.93	(10.05)	1.02	(0.16)
		Greedy Determinant	0.59	(0.12)	103302.21	(113465.69)	47.38	(10.10)	1.01	(0.16)
		MIP Trace	0.60	(0.13)	100306.17	(110342.56)	47.61	(10.13)	1.02	(0.16)
		Block R^2	0.59	(0.13)	102397.56	(112608.55)	47.34	(10.03)	1.02	(0.16)
		Mean Variances	0.70	(0.10)	32375.26	(25187.86)	40.49	(6.15)	1.21	(0.07)
		Mean Loadings	0.76	(0.13)	23204.83	(20350.70)	37.38	(6.07)	1.28	(0.08)
		Random	1.08	(0.15)	3664.78	(2745.14)	26.08	(3.60)	1.55	(0.07)
Ordered	Population	Greedy Variances	0.47	(0.10)	113378.91	(113058.68)	72.80	(16.35)	1.29	(0.04)
		Greedy Determinant	0.47	(0.10)	116284.96	(115650.58)	73.85	(16.43)	1.29	(0.04)
		MIP Trace	0.49	(0.11)	97344.28	(94488.38)	71.47	(15.53)	1.31	(0.04)
		Block R^2	0.47	(0.11)	114680.87	(114457.24)	73.95	(16.41)	1.29	(0.04)
		Mean Variances	0.55	(0.09)	46136.51	(32617.04)	63.12	(9.67)	1.40	(0.05)
		Mean Loadings	0.48	(0.11)	109448.01	(111230.69)	73.28	(16.81)	1.30	(0.04)
		Random	0.70	(0.15)	12354.86	(10673.69)	44.04	(7.66)	1.59	(0.04)
	Screening	Greedy Variances	0.55	(0.10)	144792.40	(129489.35)	53.34	(9.91)	1.21	(0.07)
		Greedy Determinant	0.56	(0.10)	161263.96	(147977.49)	55.80	(11.30)	1.19	(0.08)
		MIP Trace	0.64	(0.07)	122341.09	(99355.00)	57.07	(12.21)	1.22	(0.07)
		Block R^2	0.56	(0.10)	156685.84	(143405.09)	55.13	(10.76)	1.20	(0.08)
		Mean Variances	0.69	(0.07)	39559.25	(20585.05)	46.04	(6.00)	1.39	(0.06)
		Mean Loadings	0.64	(0.10)	74142.62	(58302.19)	49.38	(8.55)	1.32	(0.06)
		Random	0.86	(0.12)	12149.41	(8422.47)	33.60	(4.73)	1.58	(0.05)

Note. MIP = Mixed Integer Programming. Standard deviations are given in parentheses.

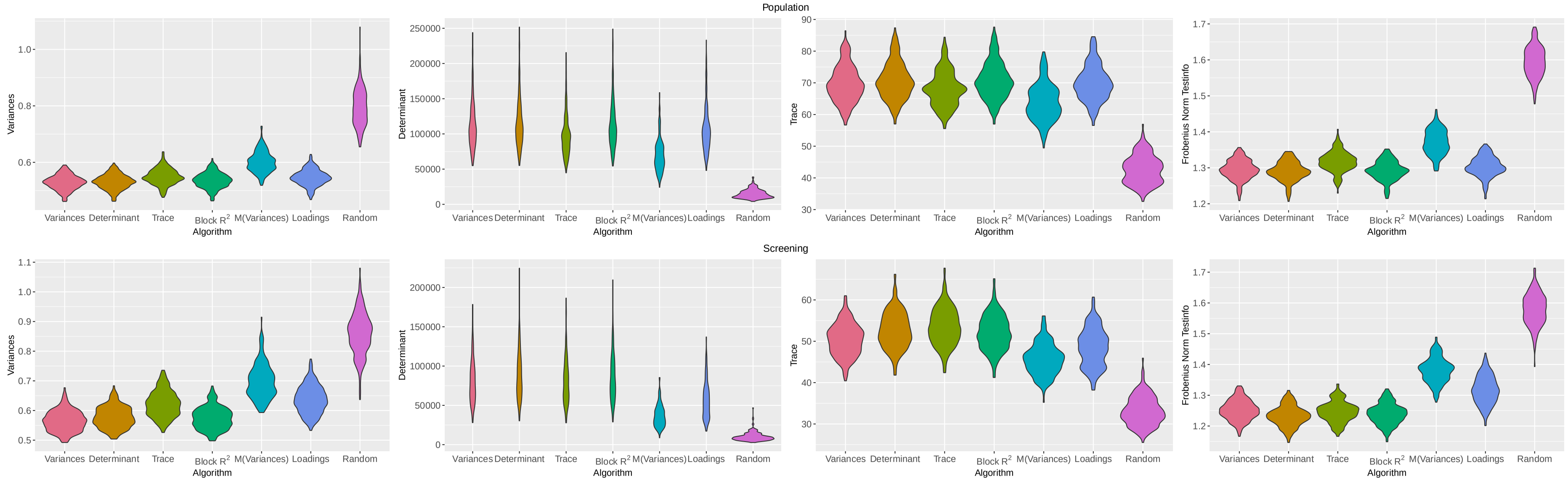

Figure S4. Optimization criteria by algorithm, for a block size of three and the ordered intercepts, in Simulation Study 2 on test construction. The bulge indicates the density, obtained by kernel density estimation. M = Mean.

Simulation on Test Construction with all Positively Keyed Items

Simulation Study 2 on test construction was replicated with all positively keyed items. Besides that, the simulation design and procedure were identical to the simulations reported in the main text.

Table S6

Variance in optimization criteria explained in % by algorithm, target, intercepts and block size in the simulation on test construction with all positively keyed items

Factor	Var.	Det.	Trace	Frob.
Algorithm vs. Random	22	3	16	41
Info vs. Mean Loadings	2	0	0	0
Algorithm vs. Means	2	3	5	12
R^2 vs. Mean Variances	3	0	1	0
Population vs. Screening	3	15	0	3
Weighted vs. Equal	5	0	0	0
2 vs. 3 and 4	18	13	22	3
3 vs. 4	5	1	3	0
Intercepts	25	9	34	11
Algorithm vs. Random × Population vs. Screening	1	2	0	2
Algorithm vs. Random × 2 vs. 3 and 4	0	2	1	1
Algorithm vs. Means × 2 vs. 3 and 4	0	2	2	2
Population vs. Screening × 2 vs. 3 and 4	0	10	0	2
Info vs. Loadings × Intercepts	0	0	1	2
Population vs. Screening × Intercepts	0	8	0	0
2 vs. 3 and 4 × Intercepts	0	4	1	3
Population × 2 vs. 3 and 4 × Intercepts	0	4	0	0
Residuals	7	8	7	8

Note. Var = Variances, Det = Determinant, Frob = Frobenius.

Table S7

Mean trait recovery by condition in the simulation on test construction with all positively keyed items

Intercepts	Target	Algorithm	Variances		Determinant		Trace		Frobenius Norm	
Random	Population	Greedy Variances	1.12	(0.12)	14187.25	(10331.67)	47.12	(10.18)	1.13	(0.08)
		Greedy Determinant	1.14	(0.11)	16737.54	(12836.44)	49.38	(11.46)	1.11	(0.09)
		MIP Trace	1.18	(0.11)	12889.82	(9425.90)	46.56	(9.90)	1.15	(0.08)
		Block R^2	1.16	(0.13)	8964.47	(6218.93)	42.45	(8.70)	1.22	(0.07)
		Mean Variances	1.32	(0.06)	4431.51	(1506.72)	39.71	(5.00)	1.31	(0.06)
		Mean Loadings	1.26	(0.08)	8740.19	(5123.23)	42.21	(7.21)	1.21	(0.05)
		Random	1.46	(0.10)	1833.80	(909.47)	28.58	(3.84)	1.46	(0.05)
	Screening	Greedy Variances	1.07	(0.14)	31463.21	(29859.46)	46.31	(10.29)	1.05	(0.14)
		Greedy Determinant	1.09	(0.14)	36621.05	(35920.82)	48.40	(11.29)	1.03	(0.15)
		MIP Trace	1.11	(0.14)	35258.72	(34536.16)	48.72	(11.37)	1.05	(0.15)
		Block R^2	1.10	(0.16)	23239.49	(22934.53)	42.95	(9.93)	1.14	(0.14)
		Mean Variances	1.15	(0.14)	27860.56	(26451.53)	46.88	(10.65)	1.10	(0.13)
		Mean Loadings	1.26	(0.09)	11773.14	(8814.14)	40.36	(6.83)	1.23	(0.07)
		Random	1.51	(0.13)	2026.96	(1303.49)	26.76	(3.81)	1.51	(0.06)
Ordered	Population	Greedy Variances	0.97	(0.12)	25431.68	(18075.25)	65.99	(11.20)	1.26	(0.03)
		Greedy Determinant	1.01	(0.10)	35278.97	(28731.16)	73.59	(16.06)	1.22	(0.03)
		MIP Trace	1.04	(0.10)	30048.43	(24000.75)	71.28	(15.62)	1.25	(0.04)
		Block R^2	1.02	(0.12)	9787.69	(5448.28)	50.49	(4.78)	1.40	(0.05)
		Mean Variances	1.15	(0.09)	10358.95	(5405.74)	59.89	(7.60)	1.38	(0.08)
		Mean Loadings	1.06	(0.10)	31927.01	(26366.17)	73.29	(16.83)	1.24	(0.04)
		Random	1.20	(0.13)	5356.36	(3867.18)	44.02	(7.65)	1.49	(0.04)
	Screening	Greedy Variances	0.89	(0.11)	114724.83	(86251.32)	63.73	(10.97)	1.21	(0.04)
		Greedy Determinant	0.94	(0.09)	169445.86	(146261.95)	70.81	(15.23)	1.16	(0.06)
		MIP Trace	0.97	(0.08)	158825.96	(135488.92)	71.55	(15.60)	1.18	(0.06)
		Block R^2	0.94	(0.11)	40176.56	(22913.19)	51.13	(6.27)	1.38	(0.06)
		Mean Variances	1.01	(0.08)	105175.56	(80158.33)	67.34	(13.32)	1.24	(0.05)
		Mean Loadings	1.00	(0.08)	146584.40	(128417.19)	70.34	(15.89)	1.20	(0.06)
		Random	1.18	(0.13)	15759.98	(11801.97)	41.90	(7.47)	1.54	(0.06)

Note. MIP = Mixed Integer Programming. Standard deviations are given in parentheses.

Table S8

Mean trait recovery by condition in the simulation on test construction with all positively keyed items for the weighted and equal target (population test)

Intercepts	Target	Algorithm	$r(\theta, \hat{\theta})$		$r(\theta, \hat{\theta})^2$		MAB		RMSE	
Random	Weighted	Greedy Variances	0.87	(0.02)	0.75	(0.03)	0.39	(0.02)	0.25	(0.03)
		Greedy Determinant	0.86	(0.02)	0.75	(0.04)	0.40	(0.03)	0.26	(0.03)
		MIP Trace	0.86	(0.02)	0.74	(0.04)	0.40	(0.03)	0.26	(0.04)
		Block R^2	0.86	(0.02)	0.75	(0.04)	0.40	(0.03)	0.25	(0.04)
		Mean Variances	0.84	(0.04)	0.71	(0.06)	0.42	(0.05)	0.29	(0.06)
		Mean Loadings	0.85	(0.02)	0.73	(0.04)	0.41	(0.03)	0.27	(0.04)
		Random	0.83	(0.03)	0.69	(0.05)	0.44	(0.04)	0.31	(0.05)
	Equal	Greedy Variances	0.82	(0.03)	0.67	(0.04)	0.38	(0.02)	0.23	(0.03)
		Greedy Determinant	0.81	(0.03)	0.66	(0.04)	0.39	(0.02)	0.23	(0.03)
		MIP Trace	0.81	(0.03)	0.65	(0.05)	0.39	(0.03)	0.24	(0.03)
		Block R^2	0.81	(0.03)	0.66	(0.05)	0.39	(0.03)	0.23	(0.03)
		Mean Variances	0.78	(0.05)	0.62	(0.08)	0.41	(0.04)	0.26	(0.05)
		Mean Loadings	0.79	(0.03)	0.63	(0.05)	0.40	(0.03)	0.25	(0.03)
		Random	0.76	(0.04)	0.58	(0.06)	0.43	(0.03)	0.29	(0.04)
Ordered	Weighted	Greedy Variances	0.87	(0.02)	0.77	(0.03)	0.38	(0.02)	0.24	(0.03)
		Greedy Determinant	0.87	(0.02)	0.75	(0.04)	0.39	(0.02)	0.25	(0.04)
		MIP Trace	0.86	(0.02)	0.75	(0.04)	0.40	(0.02)	0.26	(0.04)
		Block R^2	0.88	(0.02)	0.77	(0.04)	0.38	(0.03)	0.23	(0.03)
		Mean Variances	0.85	(0.04)	0.72	(0.07)	0.42	(0.05)	0.28	(0.07)
		Mean Loadings	0.86	(0.03)	0.74	(0.04)	0.40	(0.03)	0.27	(0.04)
		Random	0.85	(0.03)	0.73	(0.04)	0.41	(0.03)	0.27	(0.04)
	Equal	Greedy Variances	0.84	(0.02)	0.70	(0.03)	0.36	(0.02)	0.21	(0.02)
		Greedy Determinant	0.83	(0.02)	0.69	(0.03)	0.37	(0.02)	0.21	(0.02)
		MIP Trace	0.82	(0.02)	0.68	(0.04)	0.37	(0.02)	0.22	(0.03)
		Block R^2	0.84	(0.03)	0.70	(0.05)	0.36	(0.03)	0.20	(0.03)
		Mean Variances	0.81	(0.04)	0.66	(0.07)	0.39	(0.03)	0.23	(0.04)
		Mean Loadings	0.82	(0.03)	0.67	(0.04)	0.38	(0.02)	0.23	(0.03)
		Random	0.80	(0.03)	0.65	(0.05)	0.40	(0.03)	0.24	(0.04)

Note. MAB = Mean Absolute Bias, RMSE = Root Mean Squared Error, MIP = Mixed Integer Programming. Standard deviations are given in parentheses.

Table S9

Variance in trait recovery explained in % by algorithm, target and intercepts in the simulation on test construction with all positively keyed items for the weighted and equal target (population test)

Factor	$r(\theta, \hat{\theta})$	MAB	RMSE
Algorithm vs. Random	4	8	6
Info vs. Loadings	1	1	1
Algorithm vs. Means	1	1	1
R^2 vs. Mean Variances	3	4	4
Intercepts	4	6	5
Target	38	6	10
2 vs. 3 and 4	0	1	0
3 vs. 4	2	2	2
Algorithm vs. Random × Intercepts	0	1	1
Target × Intercepts	0	1	1
Algorithm vs. Random × 2 vs. 3 and 4	0	0	1
R^2 vs. Mean Variances × 2 vs. 3 and 4	2	3	3
2 vs. 3 and 4 × Intercepts	1	1	1
Residuals	42	62	61

Note. MAB = Mean Absolute Bias, RMSE = Root Mean Squared Error. $r(\theta, \hat{\theta})$ was Fisher Z transformed.

Table S10

Mean sensitivity and specificity by condition in the simulation study on test construction with all positively keyed items for the screening test

Intercepts	Algorithm	Sensitivity		Specificity	
Random	Greedy Variances	0.58	(0.07)	0.96	(0.01)
	Greedy Determinant	0.58	(0.07)	0.97	(0.01)
	MIP Trace	0.57	(0.07)	0.97	(0.01)
	Block R^2	0.58	(0.08)	0.96	(0.01)
	Mean Variances	0.56	(0.07)	0.96	(0.01)
	Mean Loadings	0.56	(0.07)	0.96	(0.01)
	Random	0.52	(0.08)	0.96	(0.01)
Ordered	Greedy Variances	0.59	(0.06)	0.97	(0.01)
	Greedy Determinant	0.58	(0.06)	0.97	(0.01)
	MIP Trace	0.57	(0.07)	0.97	(0.01)
	Block R^2	0.59	(0.07)	0.96	(0.01)
	Mean Variances	0.55	(0.07)	0.97	(0.01)
	Mean Loadings	0.57	(0.07)	0.97	(0.01)
	Random	0.55	(0.07)	0.96	(0.01)

Note. MIP = Mixed Integer Programming. Standard deviations are given in parentheses.

Table S11

Variance in sensitivity and specificity explained in % by algorithm, intercepts and block size in the simulation on test construction with all positively keyed items for the screening test

Factor	Sens.	Spec.
Algorithm vs. Random	3	0
Block R^2 vs. Mean Variances	1	0
2 vs. 3 and 4	1	2
3 vs. 4	1	0
Block R^2 vs. Mean Variances × 2 vs. 3 and 4	1	0
Algorithm vs. Random × Intercepts	1	0
2 vs. 3 and 4 × Intercepts	1	0
Residuals	89	97

Note. Sens = Sensitivity, Spec = Specificity.

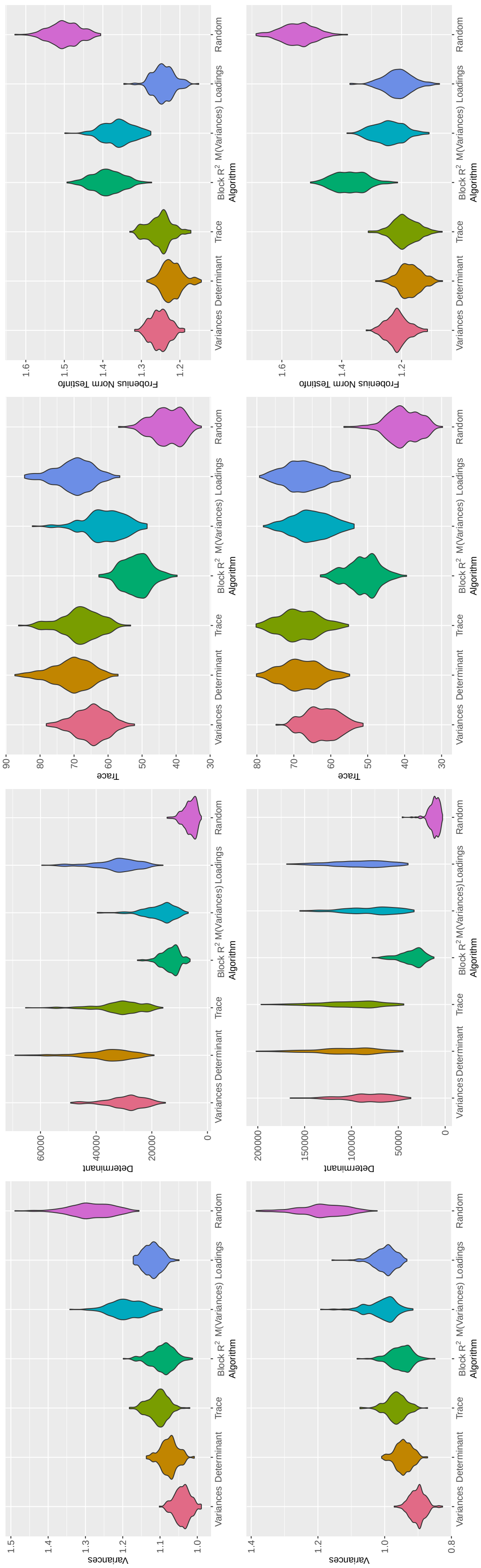

Figure S5. Optimization criteria by algorithm, for a block size of three and the ordered intercepts, in the simulation on test construction with all positively keyed items. The bulge indicates the density, obtained by kernel density estimation. M = Mean.

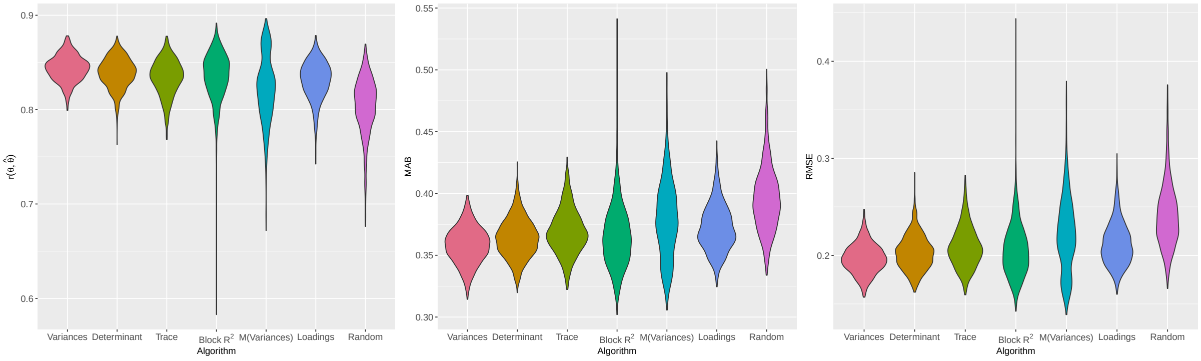

Figure S6. Trait recovery by algorithm, for a block size of three and the ordered intercepts and the equal target, in the simulation on test construction with all positively keyed items. The bulge indicates the density, obtained by kernel density estimation. M = Mean.

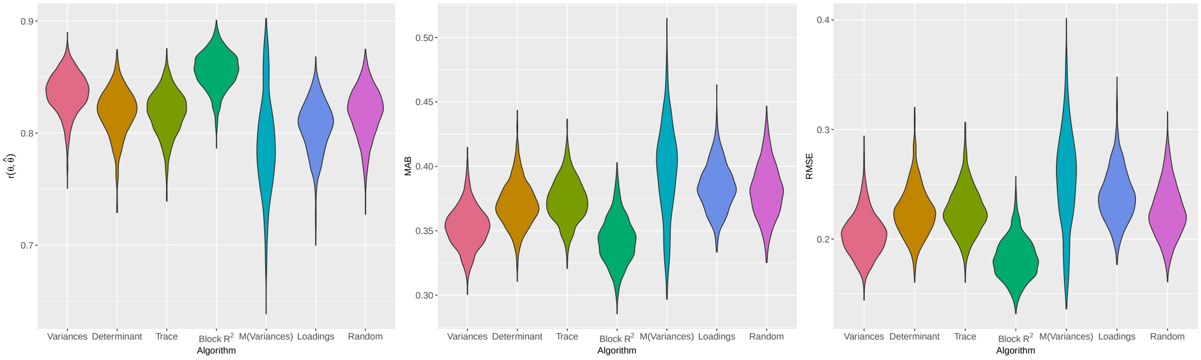

Figure S7. Trait recovery by algorithm, for a block size of two and the ordered intercepts and the equal target, in the simulation on test construction with all positively keyed items. The bulge indicates the density, obtained by kernel density estimation. M = Mean.

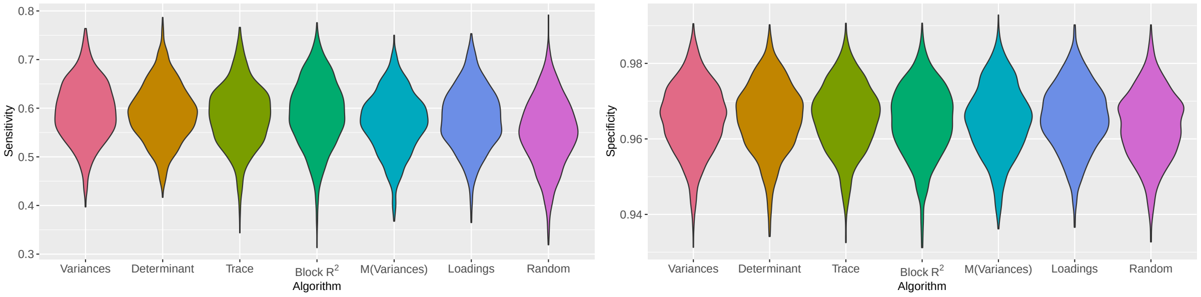

Figure S8. Sensitivity and specificity by algorithm, for a block size of three, the ordered intercepts, and the single target (screening test), in the simulation on test construction with all positively keyed items. The bulge indicates the density, obtained by kernel density estimation. M = Mean.

Empirical Application

Table S12

Item Content, Parameter Estimates, Block Information Summaries and Selected Blocks in the Big Five Inventory 2 MFC version

Block	Item Content	Trait	Key	Mixed	Item Parameters			Information Summaries			Selection		
					Load.	Pair	Int.	T-opt	R^2	Load.	T-opt	R^2	Load.
1	Is outgoing, sociable.	E	+	1	0.46	1-2	-1.74	18.5	0.07	0.39	1	1	0
	Is compassionate, has a soft heart.	A	+		0.28	1-3	0.59						
	Tends to be disorganized.	C	-		-0.41	2-3	2.42						
2	Is dependable, steady.	C	+	1	0.49	1-2	1.57	13.93	0.05	0.45	0	0	1
	Has an assertive personality.	E	+		0.47	1-3	2.03						
	Tends to find fault with others.	A	-		-0.38	2-3	0.46						
3	Is respectful, treats others with respect.	A	+	1	0.48	1-2	1.91	15.74	0.04	0.36	1	1	0
	Is relaxed, handles stress well.	N	+		-0.48	1-3	2.70						
	Rarely feels excited or eager.	E	-		-0.13	2-3	0.78						
4	Stays optimistic after experiencing a setback.	N	+	1	-0.38	1-2	0.76	5.8	0.02	0.42	0	1	1
	Is full of energy.	E	+		0.45	1-3	1.53						
	Feels little sympathy for others.	A	-		-0.42	2-3	0.89						
5	Has few artistic interests.	O	+	1	-0.46	1-2	-0.14	11.23	0.05	0.61	1	0	1
	Is dominant, acts as a leader.	E	+		0.69	1-3	0.63						
	Starts arguments with others.	A	-		-0.69	2-3	0.71						
6	Is curious about many different things.	O	+	1	0.47	1-2	0.48	8.48	0.04	0.42	0	0	0
	Has a forgiving nature.	A	+		0.48	1-3	0.99						
	Is less active than other people.	E	-		-0.31	2-3	0.59						
7	Tends to be lazy.	C	-	0	-0.50	1-2	-0.89	17.9	0.05	0.56	1	0	1
	Is sometimes shy, introverted.	E	-		-0.69	1-3	-0.21						
	Is moody, has up and down mood swings.	N	-		0.51	2-3	0.72						
8	Keeps their emotions under control.	N	+	1	-0.49	1-2	0.92	12.06	0.03	0.31	0	0	0
	Finds it hard to influence people.	E	-		0.04	1-3	-0.02						
	Is systematic, likes to keep things in order.	C	+		0.41	2-3	-0.88						
9	Has difficulty getting started on tasks.	C	-	1	-0.49	1-2	-0.29	17.39	0.06	0.56	1	1	1
	Is inventive, finds clever ways to do things.	O	+		0.44	1-3	-0.33						
	Tends to be quiet.	E	-		-0.74	2-3	-0.02						

— continued —

Block	Item Content	Trait	Key	Mixed	Item Parameters			Information Summaries			Selection		
					Load.	Pair	Int.	T-opt	R^2	Load.	T-opt	R^2	Load.
10	Is talkative.	E	+	1	0.49	1-2	0.28	18.3	0.08	0.42	1	1	0
	Can be somewhat careless.	C	-		-0.35	1-3	-0.24						
	Is fascinated by art, music, or literature.	O	+		0.40	2-3	-0.64						
11	Prefers to have others take charge.	E	-	0	-0.42	1-2	0.70	8.57	0.02	0.37	0	0	0
	Avoids intellectual, philosophical discussions.	O	-		-0.30	1-3	-0.25						
	Worries a lot.	N	-		0.38	2-3	-0.99						
12	Is complex, a deep thinker.	O	+	0	0.37	1-2	0.86	13.65	0.04	0.51	0	1	1
	Shows a lot of enthusiasm.	E	+		0.48	1-3	0.90						
	Is emotionally stable, not easily upset.	N	+		-0.67	2-3	0.15						
13	Is helpful and unselfish with others.	A	+	0	0.48	1-2	0.75	16.9	0.05	0.52	0	0	0
	Keeps things neat and tidy.	C	+		0.45	1-3	0.77						
	Feels secure, comfortable with self.	N	+		-0.62	2-3	0.07						
14	Often feels sad.	N	-	1	0.48	1-2	-1.04	22.69	0.06	0.6	1	1	1
	Is efficient, gets things done.	C	+		0.72	1-3	0.98						
	Is sometimes rude to others.	A	-		-0.59	2-3	1.93						
15	Is reliable, can always be counted on.	C	+	1	0.50	1-2	1.47	14.73	0.07	0.45	0	1	0
	Values art and beauty.	O	+		0.37	1-3	1.61						
	Is suspicious of others intentions.	A	-		-0.48	2-3	0.25						
16	Leaves a mess, doesnt clean up.	C	-	1	-0.44	1-2	-1.92	6.75	0.03	0.61	0	0	1
	Is polite, courteous to others.	A	+		0.38	1-3	0.11						
	Has little creativity.	O	-		-1.02	2-3	2.07						
17	Can be cold and uncaring.	A	-	0	-0.49	1-2	-1.44	16.96	0.06	0.5	1	1	0
	Can be tense.	N	-		0.53	1-3	-0.24						
	Has difficulty imagining things.	O	-		-0.48	2-3	1.43						
18	Assumes the best about people.	A	+	1	0.48	1-2	1.02	7.75	0.03	0.44	1	0	1
	Thinks poetry and plays are boring.	O	-		-0.26	1-3	1.09						
	Rarely feels anxious or afraid.	N	+		-0.60	2-3	-0.12						

— continued —

Block	Item Content	Trait	Key	Mixed	Item Parameters			Information Summaries			Selection		
					Load.	Pair	Int.	T-opt	R^2	Load.	T-opt	R^2	Load.
19	Is persistent, works until the task is finished.	C	+	1	0.49	1-2	1.20	28.13	0.07	0.54	1	1	1
	Tends to feel depressed, blue.	N	-		0.48	1-3	1.60						
	Has little interest in abstract ideas.	O	-		-0.64	2-3	0.33						
20	Is original, comes up with new ideas.	O	+	1	0.45	1-2	0.52	13.25	0.04	0.49	0	0	0
	Is temperamental, gets emotional easily.	N	-		0.41	1-3	1.31						
	Sometimes behaves irresponsibly.	C	-		-0.61	2-3	0.82						

Note. N = Neuroticism, E = Extraversion, O = Openess, A = Agreeableness, C = Conscientiousness, Key = Item Keying, Mixed = Mixed Keyed Block, Load. = Standardized Loading, Int. = Intercept, T-opt = T-optimality, R^2 = Block R^2, M = Mean.
The table shows weighted mean T-optimality, in the Mixed Integer Programming Algorithm, T-optimality for each grid point separately was used.
Intercepts were computed for binary outcomes of pairwise comparisons, as indicated by the column Pair.

References

Brown, A., & Maydeu-Olivares, A. (2011, June). Item response modeling of forced-choice questionnaires. *Educational and Psychological Measurement*, *71*(3), 460–502. doi: 10.1177/0013164410375112

Lin, Y. (2020). Asking the Right Questions: Increasing Fairness and Accuracy of Personality Assessments with Computerised Adaptive Testing. *Doctoral Dissertation.* doi: 10.1177/0013164416646162

Yousfi, S. (2018). Considering Local Dependencies: Person Parameter Estimation for IRT Models of Forced-Choice Data. In M. Wiberg, S. Culpepper, R. Janssen, J. González, & D. Molenaar (Eds.), *Quantitative Psychology* (Vol. 233, pp. 175–181). Cham: Springer International Publishing. doi: 10.1007/978-3-319-77249-3
